# Supplementary material for: The relationship between circulating lipids and breast cancer risk: A Mendelian randomization study
Source: PLoS Med. 2020 Sep 11;17(9):e1003302. doi: 10.1371/journal.pmed.1003302 (PMC7485834; doi:10.1371/journal.pmed.1003302)
Supplement: S1 STROBE-MR checklist — MR, Mendelian randomization; STROBE, Strengthening the Reporting of Observational Studies in Epidemiology. (DOCX) [file pmed.1003302.s002.docx]

# Strobe-MR checklist

| **Item** | **Complete/location** |
| --- | --- |
| 1. **Title and Abstract:** "Mendelian randomization" is named both in the title and the abstract | **Complete** |
| **Introduction** |  |
| 1. **Background:** Explain the scientific background and rationale for the reported study. Is causality between exposure and outcome plausible? Justify why MR is a helpful method to address the study question. | In the first and second paragraphs of the introduction, we discuss why lipids are a plausible causal exposure for breast cancer. In the second and third paragraphs we discuss why Mendelian randomization is a reasonable approach to investigate a potential relationship between lipids and breast cancer. |
| 1. **Objectives:** State specific objectives clearly, including pre-specified causal hypotheses (if any). | In the 4th, 5th and 6th paragraphs of the introduction we describe the objectives of our study. |
| **Methods** |  |
| 1. **Study design and data sources:** Present key elements of study design early in the paper. Consider including a table listing sources of data for all phases of the study. For each data source contributing to the analysis, describe the following:   a) Describe the study design and the underlying population from which it was drawn. Describe also the setting, locations, and relevant dates, including periods of recruitment, exposure, follow-up, and data collection, if available.  b) Give the eligibility criteria, and the sources and methods of selection of participants.  c) Explain how the analyzed sample size was arrived at.  d) Describe measurement, quality and selection of genetic variants.  e) For each exposure, outcome and other relevant variables, describe methods of assessment and, in the case of diseases, the diagnostic criteria used.  f) Provide details of ethics committee approval and participant informed consent, if relevant. | Available information about the GWAS studies is provided in the Supplementary Methods, and the section “GWAS details”. Further information is given in each of the original GWAS publications.  Ethics approval and informed consent info in the "Study Populations" section of the method section.  Selection of genetic variants is described in "Mendelian randomization analyses" in the Methods section. |
| 1. **Assumptions:** Explicitly state assumptions for the main analysis (e.g. relevance, exclusion, independence, homogeneity) as well assumptions for any additional or sensitivity analysis. | Described in the "Mendelian randomization analyses" section of the methods |
| 1. **Statistical methods main analysis**   Describe statistical methods and statistics used.  a) Describe how quantitative variables were handled in the analyses (i.e., scale, units, model).  b) Describe the process for identifying genetic variants and weights to be included in the  analyses (i.e, independence and model). Consider a flow diagram.  c) Describe the MR estimator, e.g. two-stage least squares, Wald ratio, and related statistics.  Detail the included covariates and, in case of two-sample MR, whether the same covariate set was used for adjustment in the two samples.  d) Explain how missing data were addressed.  e) If applicable, say how multiple testing was dealt with. | (a) Described in the "Mendelian randomization analyses" section of the methods  b) Described in the "Mendelian randomization analyses" section of the methods  c) Described in the "Mendelian randomization analyses" section of the methods  d) Described in the "Mendelian randomization analyses" section of the methods  e) Described in the "Mendelian randomization analyses" section of the methods |
| 1. **Assessment of assumptions: Describe any methods used to assess the assumptions or justify their validity.** | Our test for instrument heterogeneity is described in the "Mendelian randomization analyses" section and in the "Heterogeneity analyses for single trait MR" and "Multivariable MR tests of instrument strength and validity" sections of the supplementary methods. |
| 1. **Sensitivity analyses:** Describe any sensitivity analyses or additional analyses performed. | Our test of instrument sensitivity is described in the "Mendelian randomization analyses" section of the Method section. |
| 1. **Software and pre-registration**   a) Name statistical software and package(s), including version and settings used.  b) State whether the study protocol and details were pre-registered (as well as when and  where). | a) All statistical software and settings used are described in the "Mendelian randomization analyses" and "Genetic correlation analyses" section of the Methods section.  b) The analysis plan is described in the "Analysis Plan" section of the Method section. |
| **Results** |  |
| 1. **Descriptive data**   a) Report the numbers of individuals at each stage of included studies and reasons for exclusion. Consider use of a flow-diagram.  b) Report summary statistics for phenotypic exposure(s), outcome(s) and other relevant variables (e.g. means, standard deviations, proportions).  c) If the data sources include meta-analyses of previous studies, provide the number of studies, their reported ancestry, if available, and assessments of heterogeneity across these studies. Consider using a supplementary table for each data source.  d) For two-sample Mendelian randomization:  i. Provide information on the similarity of the genetic variant-exposure associations between the exposure and outcome samples.  ii. Provide information on extent of sample overlap between the exposure and outcome data sources. | a) Information is given in the "Study Populations" section of the methods.  b) We give the summary statistics for our instruments in Supplementary tables 1-3. Summary statistics are also available from each GWAS as described in the "GWAS details" section of the Supplementary methods.  c) We give this information, when available, in the "GWAS details" section of the Supplementary Methods.  d) We provide this information in the "Study populations" section of the Method section and the "GWAS details" section of the Supplementary Methods. |
| 1. **Main results**   a) Report the associations between genetic variant and exposure, and between genetic variant and outcome, preferably on an interpretable scale (e.g. comparing 25th and 75th percentile of allele count or genetic risk score, if individual-level data available).  b) Report causal effect estimate between exposure and outcome, and the measures of uncertainty from the MR analysis. Use an intuitive scale, such as odds ratio, or relative  risk, per standard deviation difference.  c) If relevant, consider translating estimates of relative risk into absolute risk for a meaningful time-period.  d) Consider any plots to visualize results (e.g. forest plot, scatterplot of associations between genetic variants and outcome versus between genetic variants and exposure). | Our results are given in terms of odds ratio and confidence intervals throughout the results section. We visualize results using a forest plot in Figures 1 and 2 and a scatter plot of SNP effects on exposures versus outcomes in Supplementary Figure 3. |
| 1. **Assessment of assumptions**   a) Assess the validity of the assumptions.  b) Report any additional statistics (e.g., assessments of heterogeneity, such as I2, Q statistic). | a) We assess the validity using sensitivity analyses and Egger regression, described in the "Single trait Mendelian randomization (MR) in breast cancer" section of the results section.  b) We discuss the use of Cochran's Q statistic in the "Mendelian randomization analyses" section of the methods section and in our supplementary method section |
| 1. **Sensitivity and additional analyses**   a) Use sensitivity analyses to assess the robustness of the main results to violations of the assumptions.  b) Report results from other sensitivity analyses (e.g., replication study with different dataset, analyses of subgroups, validation of instrument(s), simulations, etc.).  c) Report any assessment of direction of causality (e.g., bidirectional MR).  d) When relevant, report and compare with estimates from non-MR analyses.  e) Consider any additional plots to visualize results (e.g., leave-one-out analyses). | Results section “Single trait Mendelian randomization (MR) in breast cancer”: Sensitivity analyses (paragraph 1&2), replication with genetic instruments with a separate GWAS (paragraph 2), directionality assessments (paragraph 2).  Results section “Multivariable Mendelian randomization with age at menarche and body mass index as exposures” paragraph 1: Sensitivity analyses, replication with genetic instruments with a separate GWAS. |
| **Discussion** |  |
| 1. **Key results** | We describe key results in the first paragraph of the discussion section. |
| 1. **Limitations**   Discuss limitations of the study, taking into account the validity of the MR assumptions, other sources of potential bias, and imprecision. Discuss both direction and magnitude of any potential bias, and any efforts to address them. | Discussion paragraph 5 |
| 1. **Interpretations**   a) Give a cautious overall interpretation of results considering objectives and limitations.  Compare with results from other relevant studies.  b) Discuss underlying biological mechanisms that could be modelled by using the genetic  variants to assess the relationship between the exposure and the outcome.  c) Discuss whether the results have clinical or policy relevance, and whether interventions  could have the same size effect. | a) Interpretation: Discussion paragraphs 1, 4, 5, 6, & 7; comparison with other studies: Discussion paragraphs 2 & 3.  b) Discussion paragraph 6  c) Discussion paragraph 7 |
| 1. **Generalizability:** | In the fifth paragraph of the discussion section, we discuss potential caveats in terms of generalizing our findings. |
| 1. **Funding:** | We have reported all sources of funding. In addition, grant information for the GWAS we used are given in the "GWAS details" section of the Supplementary Methods. |
| 1. **Data and data sharing:** | We give access information to all data used in the study in the "GWAS details" section of our Supplementary Methods. Links to the statistical software used, including TwoSampleMR, LD score regression and ρ-Hess are given in the "Mendelian randomization analyses" and "Genetic correlation analyses" portions of our methods section. |
| 1. **Conflicts of Interest:** | All authors have declared conflicts of interest (none reported). |
