# Supplementary figures and images for: The relationship between circulating lipids and breast cancer risk: A Mendelian randomization study
Source: PLoS Med. 2020 Sep 11;17(9):e1003302. doi: 10.1371/journal.pmed.1003302 (PMC7485834; doi:10.1371/journal.pmed.1003302)

HDL

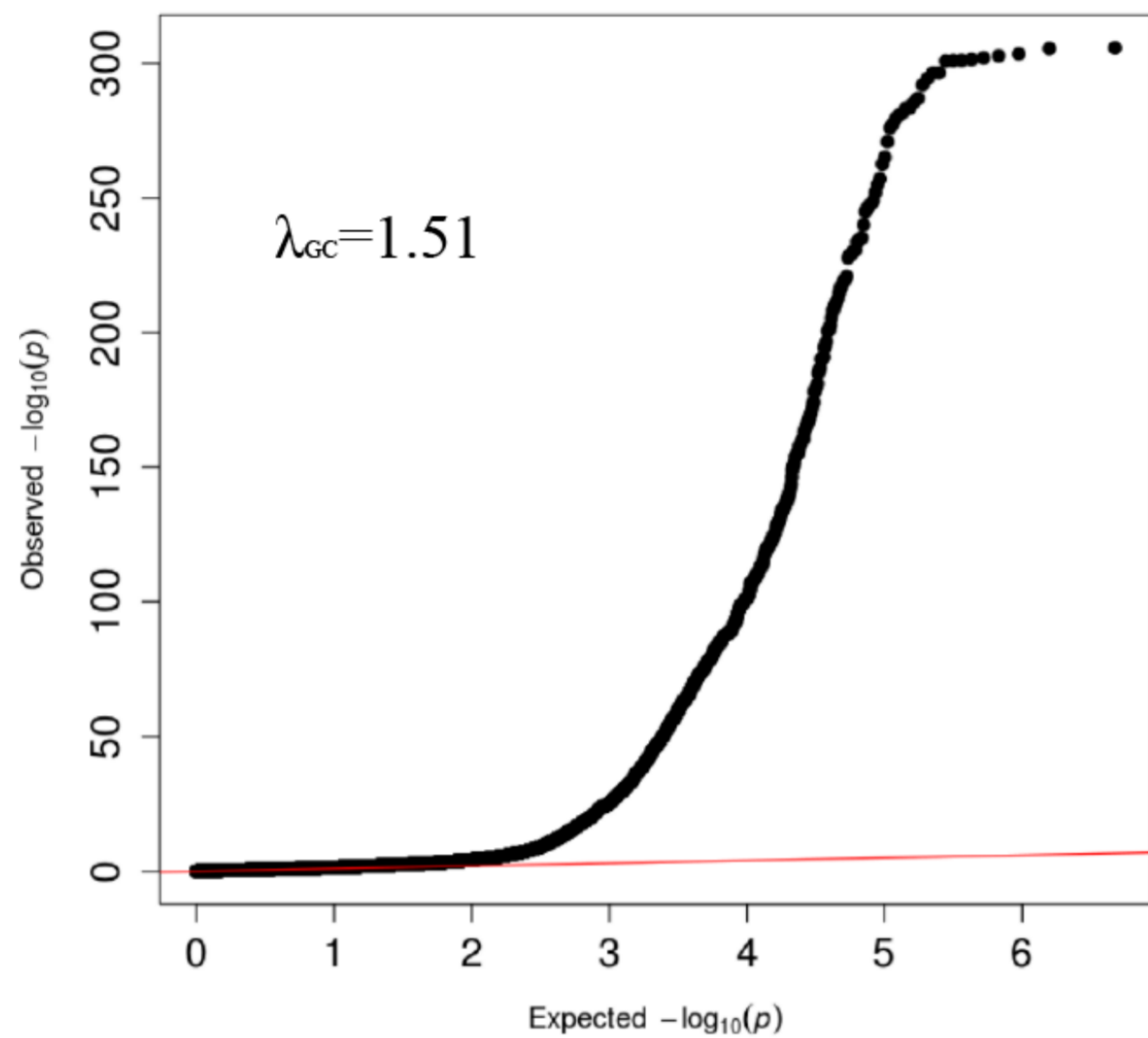

LDL

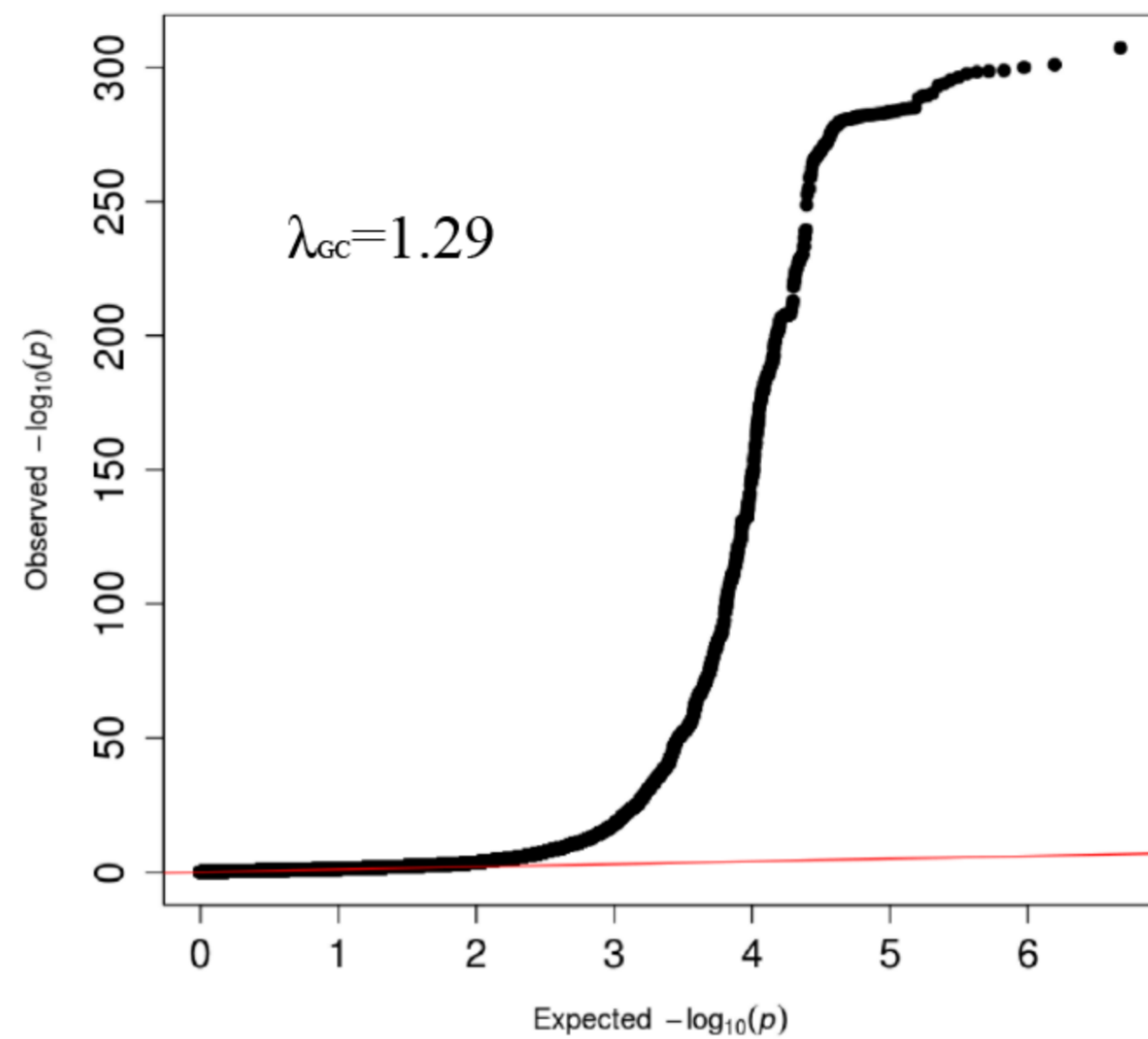

TC

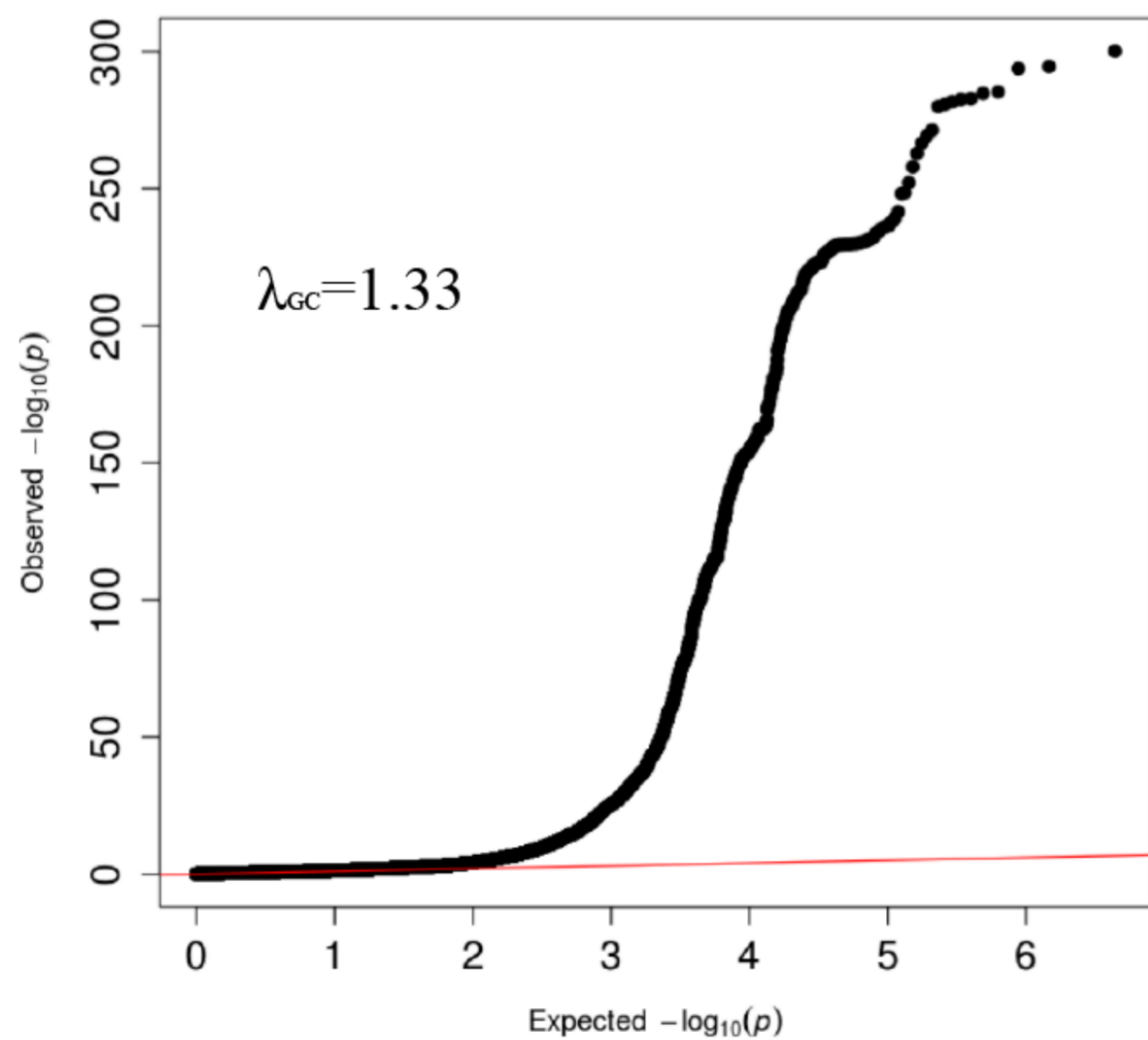

TG

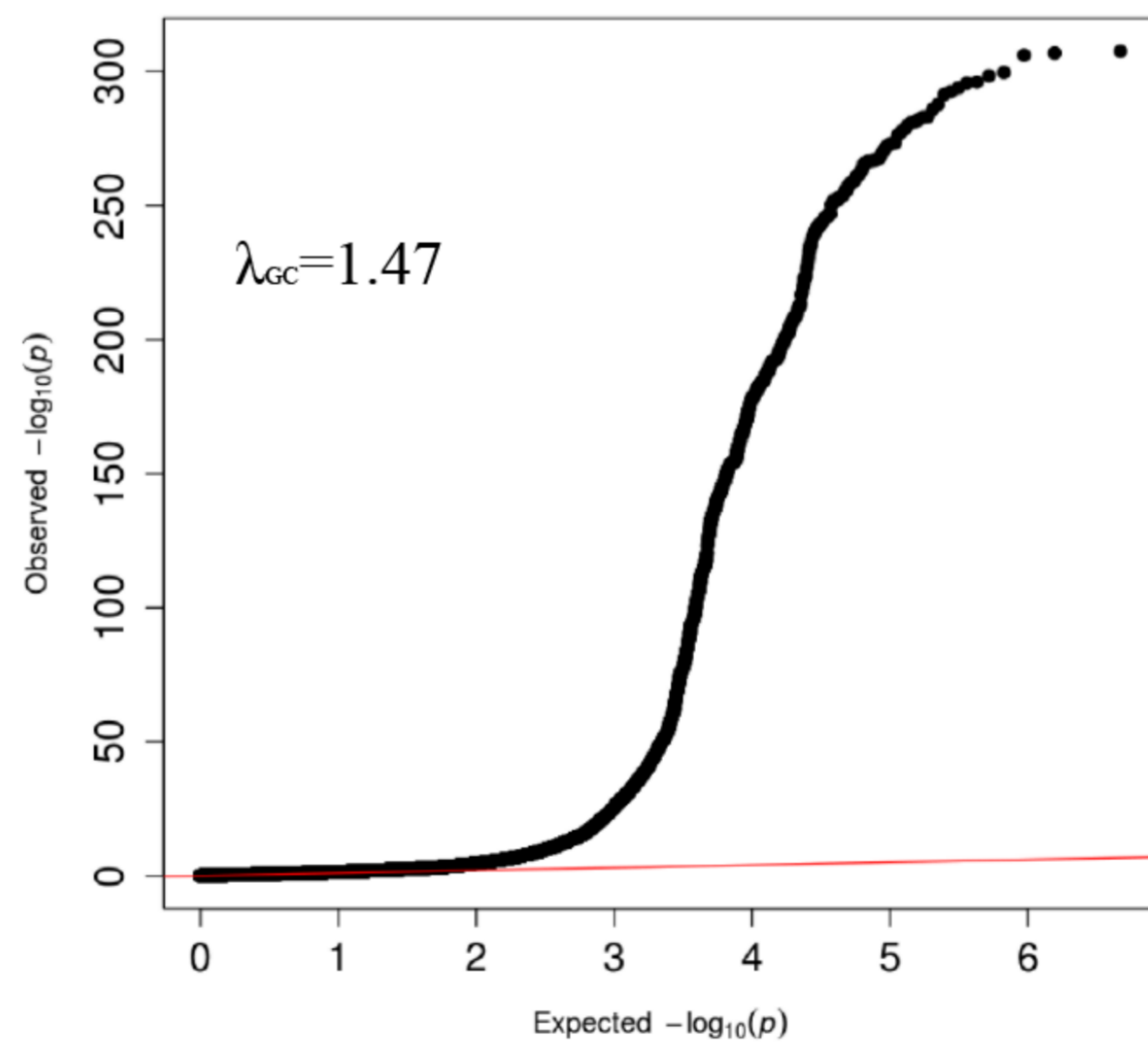

Supplement: S1 Fig — Generated from a meta-analysis of Klarin and colleagues [22] and Willer and colleagues [12]. LD-score regression intercepts and standard errors from the meta-analysis association statistics were as follows: TC, 1.1293 (0.1143); TG, 1.0317 (0.0656); LDL, 1.0933 (0.1001); HDL, 1.1715 (0.0758); see also S14 Table. HDL, high-density lipoprotein; LD, linkage disequilibrium; LDL, low-density lipoprotein; TC, total cholesterol; TG, triglyceride; λGC, genomic inflation factor. (PDF) [file pmed.1003302.s003.pdf]

Exposure

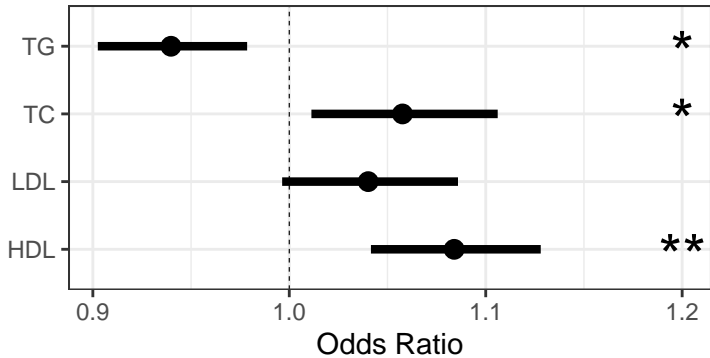

Supplement: S4 Fig — The exposure association data for this analysis were from MVP. Genetic instruments were pruned to pass heterogeneity test. Error bars represent the 95% CI. Estimates were calculated using the IVW method. *P < 0.05; **P < 0.001. See S7 Table for ORs, CIs, and P-values. BC, breast cancer; CI, confidence interval; HDL, high-density lipoprotein; IVW, inverse-variance weighted; LDL, low-density lipoprotein; MR, Mendelian randomization; MVP, Million Veteran Program; OR, odds ratio; TC, total cholesterol; TG, triglyceride. (PDF) [file pmed.1003302.s006.pdf]

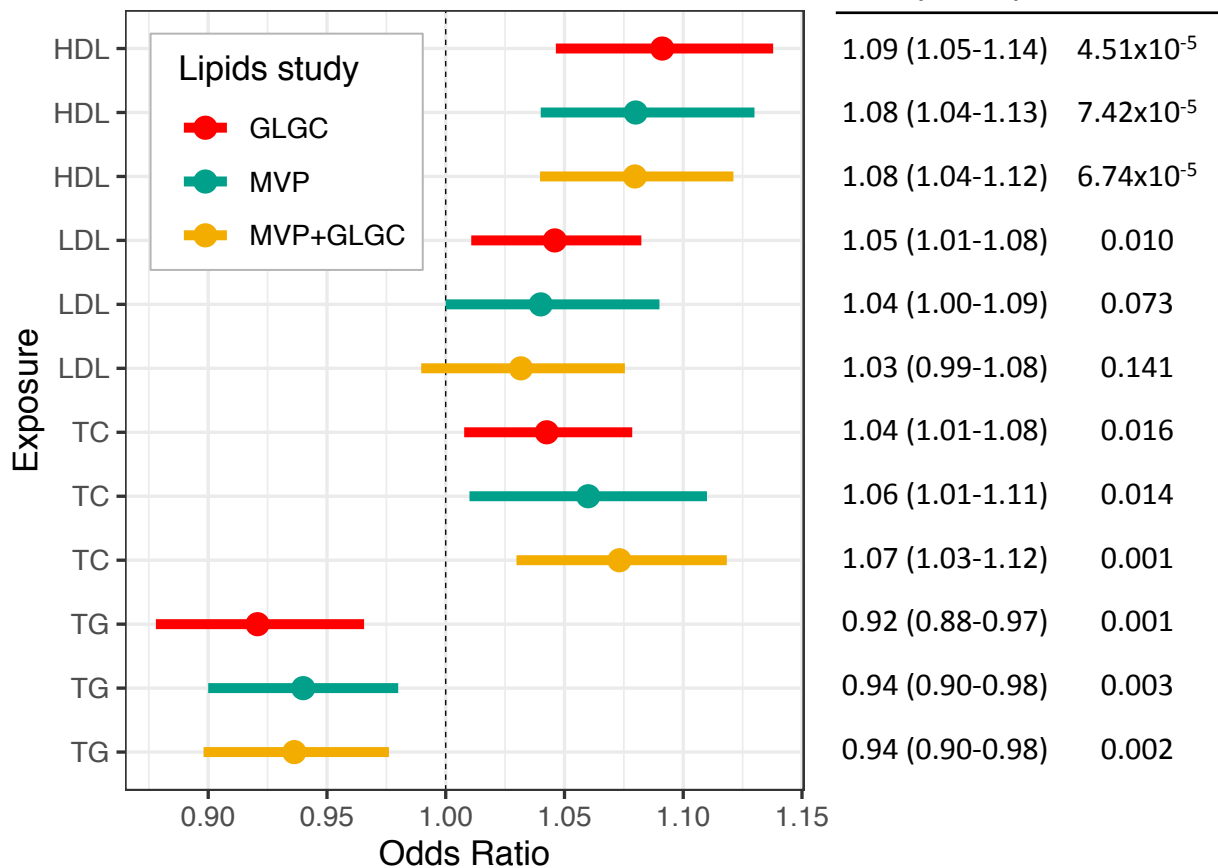

Supplement: S5 Fig — Genetic association statistics for all BCs were used for the outcome. Genetic instruments were pruned to pass heterogeneity test. Error bars represent the 95% CI. Estimates were calculated using the IVW method. BC, breast cancer; CI, confidence interval; GLGC, Global Lipids Genetics Consortium; HDL, high-density lipoprotein; IVW, inverse-variance weighted; LDL, low-density lipoprotein; MR, Mendelian randomization; MVP, Million Veteran Program; TC, total cholesterol; TG, triglyceride. (PDF) [file pmed.1003302.s007.pdf]

## Oncoarray

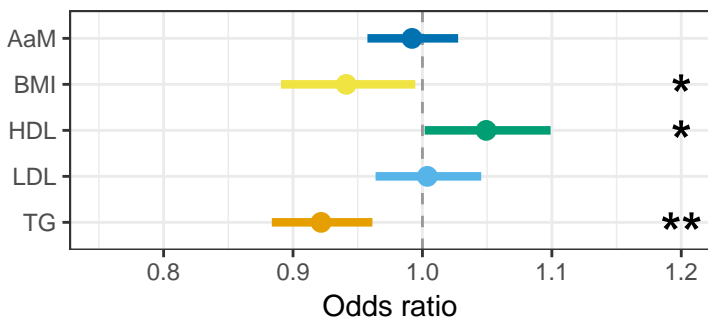

## iCOGS

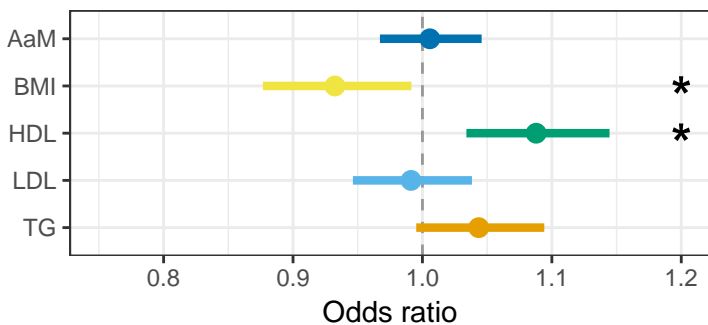

## GWAS

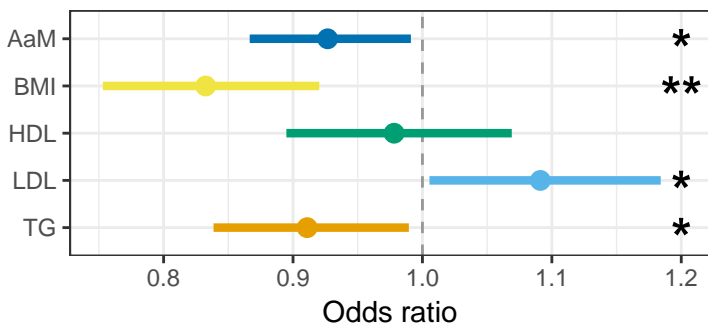

## BC meta-analysis

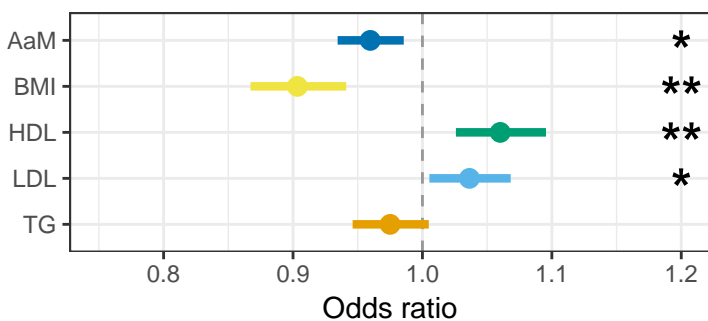

Supplement: S6 Fig — Results of multivariable MR analyses with 3 lipid traits (HDL, LDL, TG), BMI, and AaM as exposures and BC risk as the outcome. Each panel presents multivariable MR results using BC summary statistics from an independent subset of the BCAC data set (Oncoarray, iCOGS, or GWAS) or from the meta-analysis of all 3 together (BC meta-analysis, S1 Text). Results plotted are after pruning for instrument heterogeneity. Error bars represent the 95% CI. Estimates were calculated using the IVW method. *P < 0.05; **P < 0.001. See S10 Table for ORs, CIs, and P-values. AaM, age at menarche; BC, breast cancer; BCAC, Breast Cancer Association Consortium; BMI, body mass index; CI, confidence interval; GWAS, genome-wide association study; HDL, high-density lipoprotein; IVW, inverse-variance weighted; LDL, low-density lipoprotein; MR, Mendelian randomization; OR, odds ratio; TG, triglyceride. (PDF) [file pmed.1003302.s008.pdf]

**A**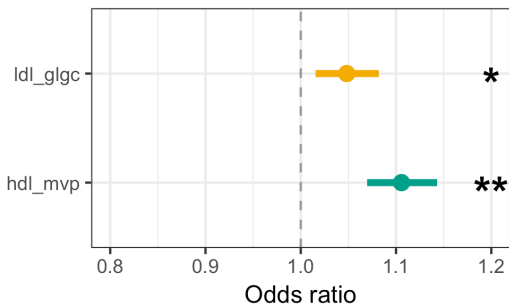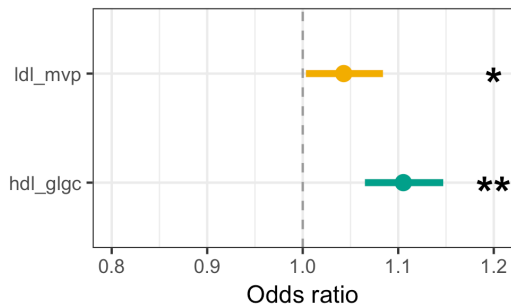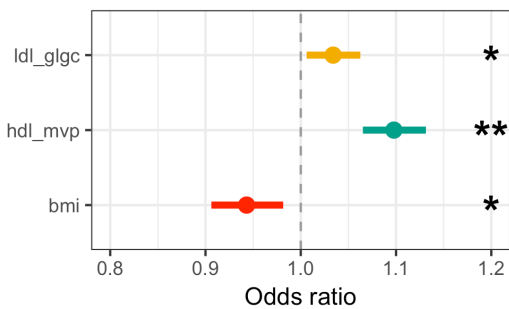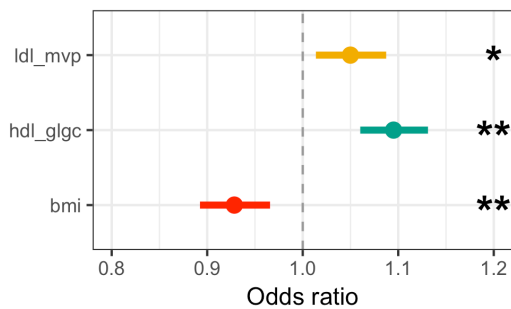**B**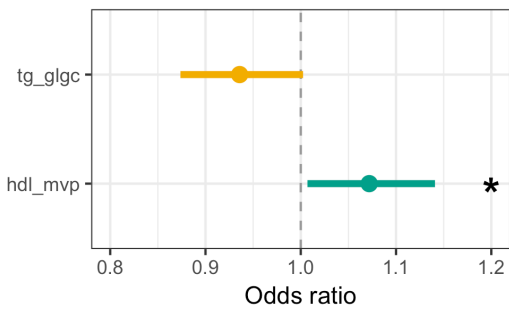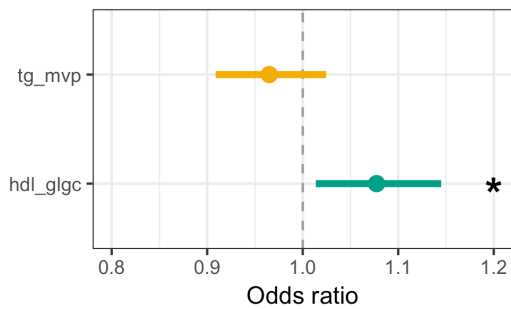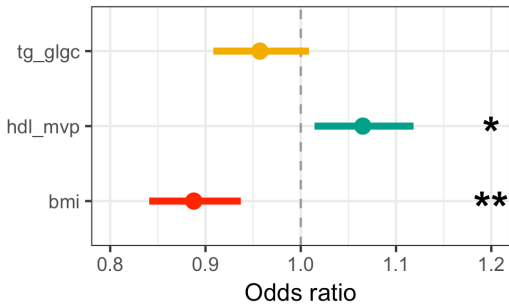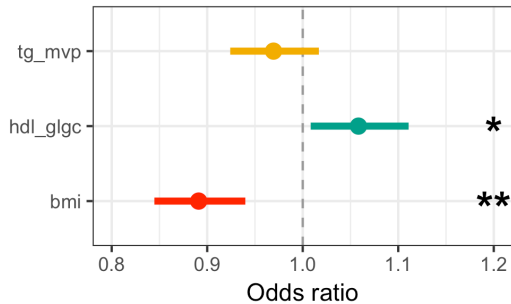

Supplement: S7 Fig — Results of multivariable MR analyses including 2 lipid traits as exposures: (A) LDL and HDL or (B) TGs and HDL, with and without BMI as an additional exposure and with risk for all BCs as the outcome. Results plotted are after pruning for instrument heterogeneity. The lipid effect estimates were from one of 2 GWAS data sets (MVP or GLGC), and the results of each combination of lipid data sets are in a single plot. Error bars represent the 95% CI. Estimates were calculated using the IVW method. *P < 0.05; **P < 0.001. See S11 Table for ORs, CIs, and P-values. BC, breast cancer; BMI, body mass index; CI, confidence interval; GLGC, Global Lipids Genetics Consortium; GWAS, genome-wide association study; HDL, high-density lipoprotein; IVW, inverse-variance weighted; LDL, low-density lipoprotein; MR, Mendelian randomization; MVP, Million Veteran Program; OR, odds ratio; TG, triglyceride. (PDF) [file pmed.1003302.s009.pdf]

Exposure

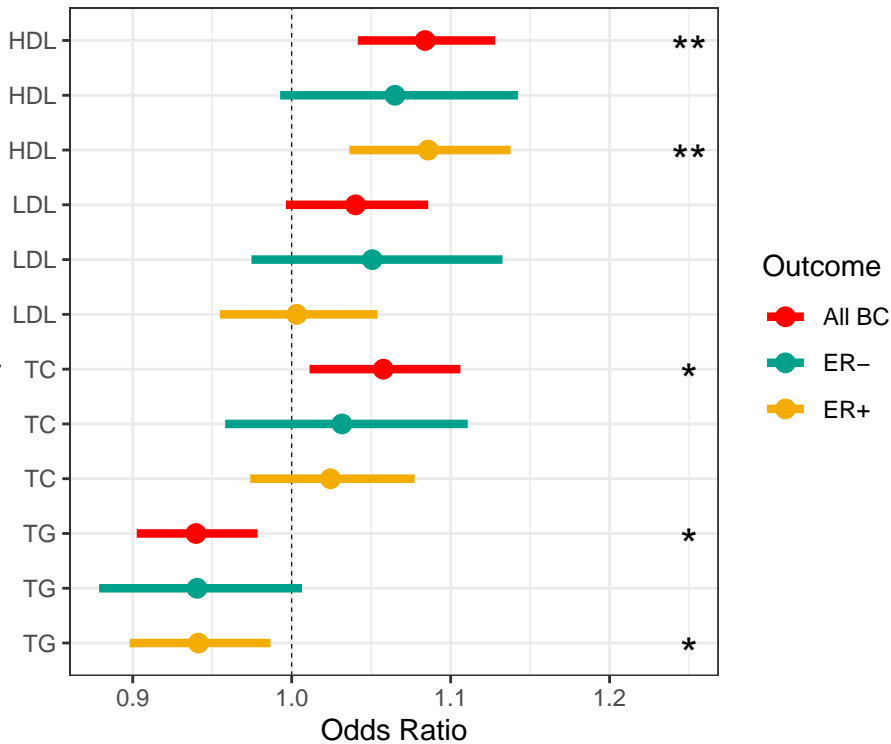

Supplement: S8 Fig — Results of single-trait MR with each lipid trait as an exposure, and one of 3 BC traits as the outcome: all BC, ER− BCs only, or ER+ BCs only. Error bars represent the 95% CI. Estimates were calculated using a fixed-effects IVW method after pruning for instrument heterogeneity. Lipid association statistics come from the MVP data. **P < 0.001, *P < 0.05. See S12 Table for ORs, CIs, and P-values. BC, breast cancer; CI, confidence interval; ER, estrogen receptor; HDL, high-density lipoprotein; IVW, inverse-variance weighted; LDL, low-density lipoprotein; MR, Mendelian randomization; OR, odds ratio; TC, total cholesterol; TG, triglyceride. (PDF) [file pmed.1003302.s010.pdf]

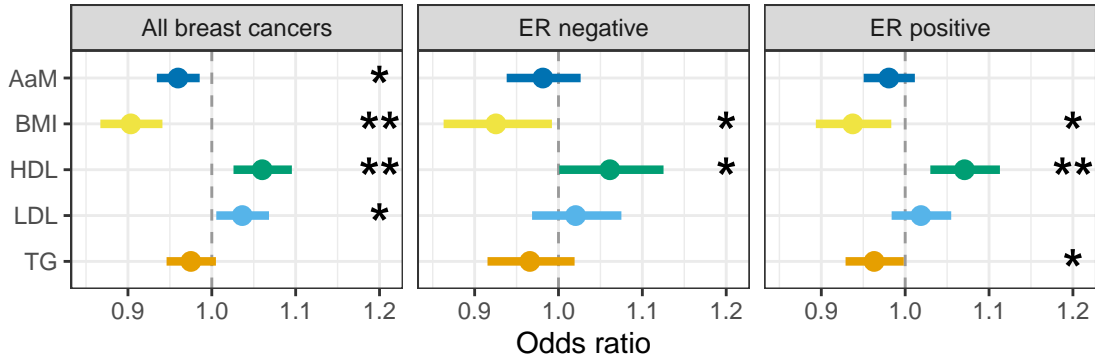

Supplement: S9 Fig — Results of multivariable MR analyses with 3 lipid traits (HDL, LDL, TGs), BMI, and AaM as exposures; and all BCs, ER‒, or ER+ BC as the outcome. Results plotted are after pruning for instrument heterogeneity. Error bars represent the 95% CI. Estimates were calculated using the IVW method. *P < 0.05; **P < 0.001. See S13 Table for ORs, CIs, and P-values. AaM, age at menarche; BC, breast cancer; BMI, body mass index; CI, confidence interval; ER, estrogen receptor; HDL, high-density lipoprotein; IVW, inverse-variance weighted; LDL, low-density lipoprotein; MR, Mendelian randomization; TG, triglyceride. (PDF) [file pmed.1003302.s011.pdf]

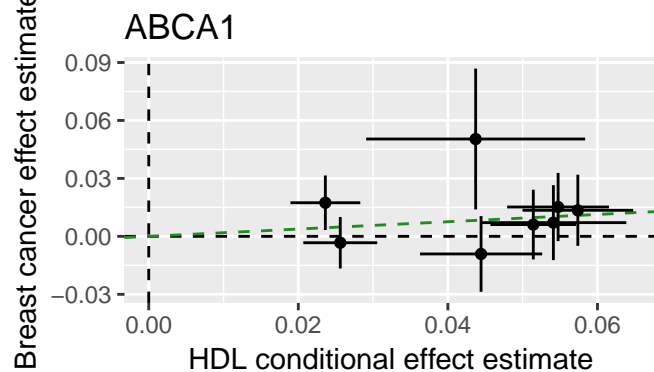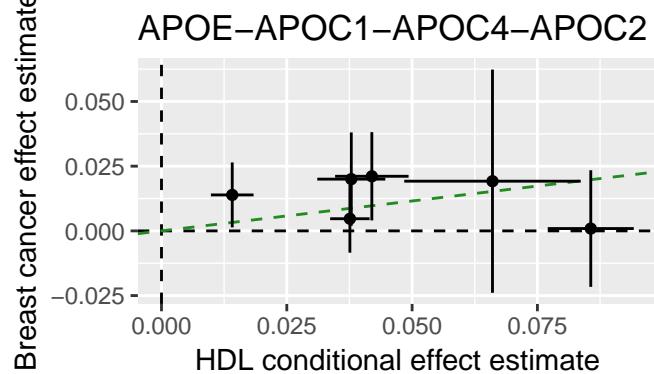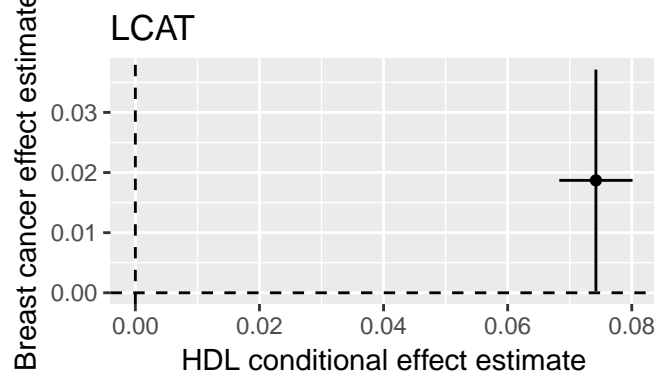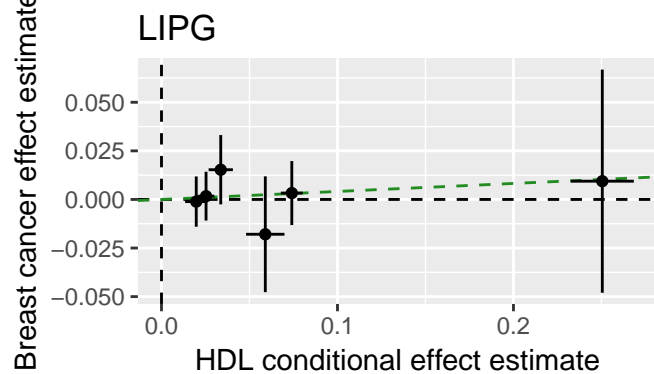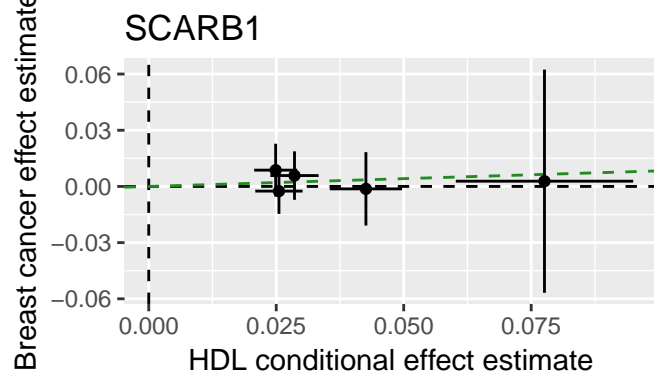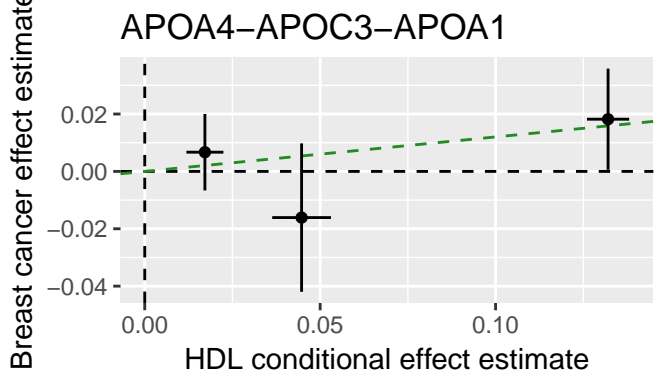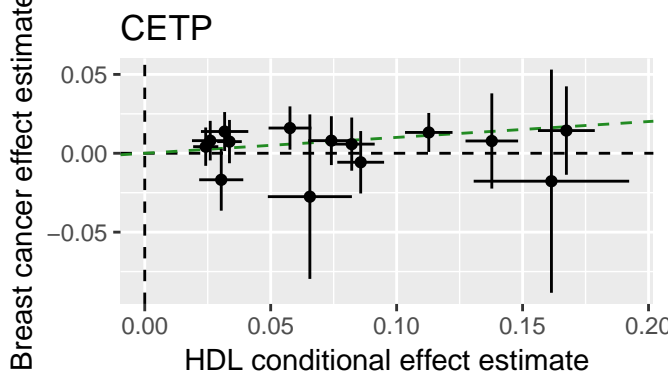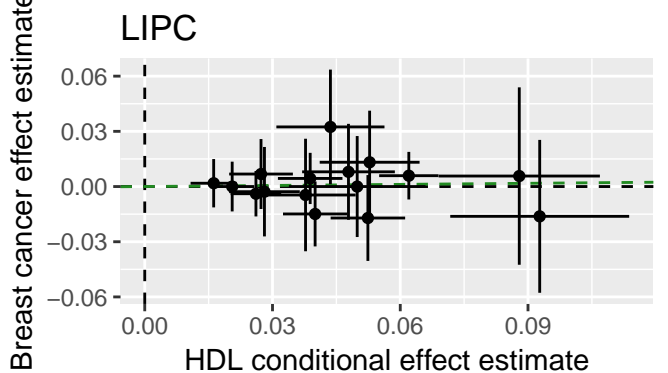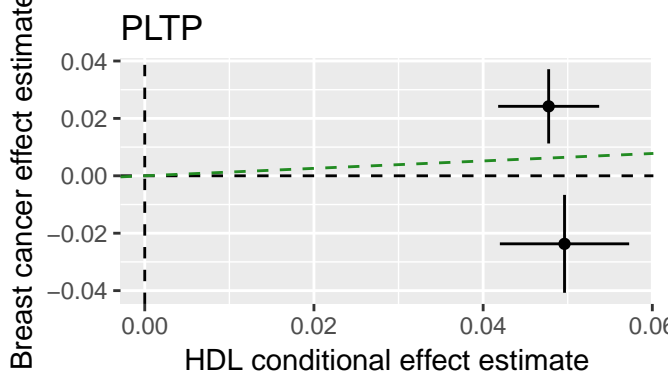

Supplement: S10 Fig — Conditionally independent HDL-associated SNPs at canonical HDL metabolism pathway genes, plotted by their conditional effect estimates on HDL (from MVP) and effect estimates on all BCs. Error bars represent 95% CIs. The dashed green line represents the regression line from fixed-effects IVW MR. BC, breast cancer; CI, confidence interval; HDL, high-density lipoprotein; IVW, inverse-variance weighted; MR, Mendelian randomization; MVP, Million Veteran Program. (PDF) [file pmed.1003302.s012.pdf]

APOB

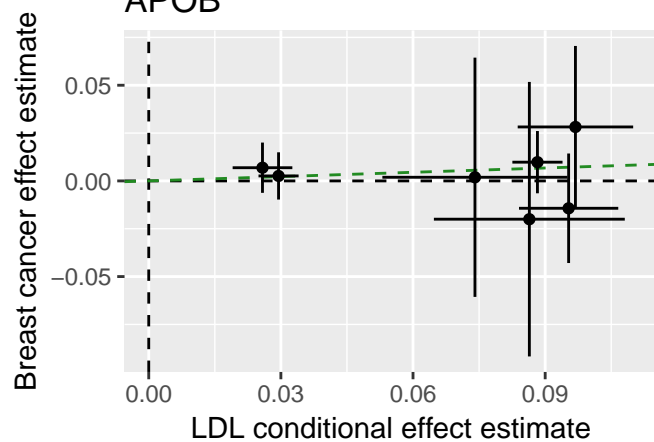

HMGCR

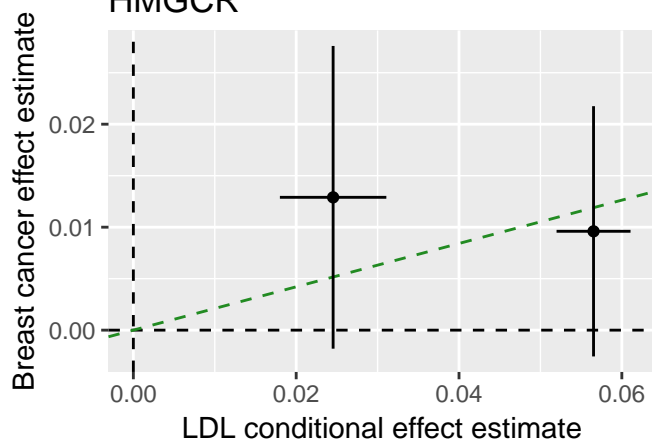

LDLR

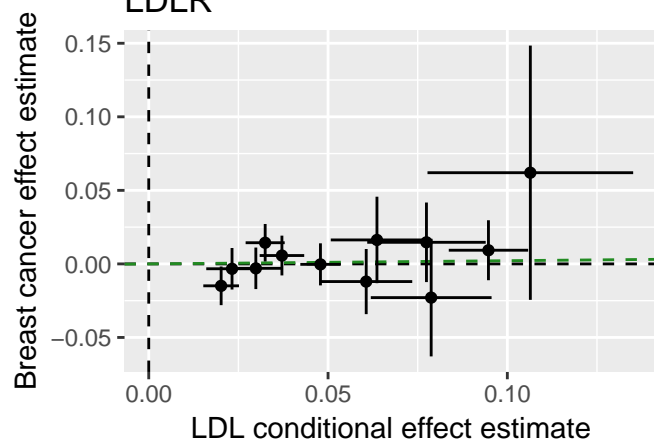

LPA

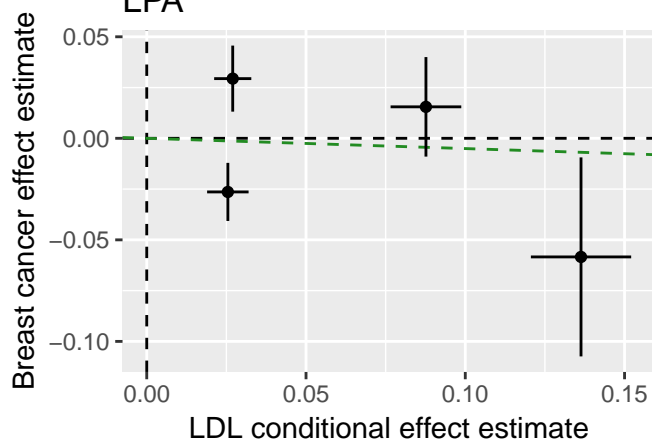

MYLIP

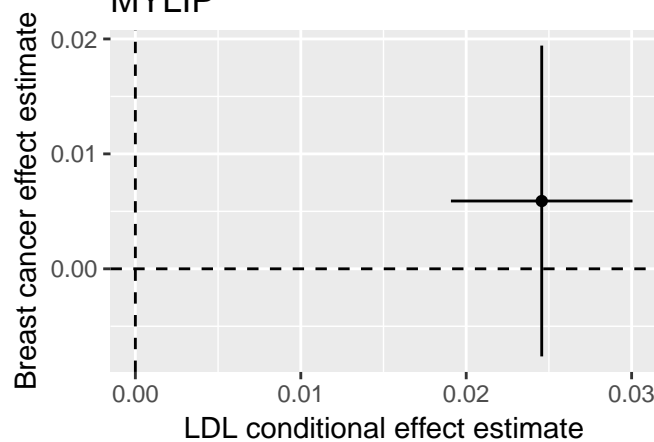

NPC1L1

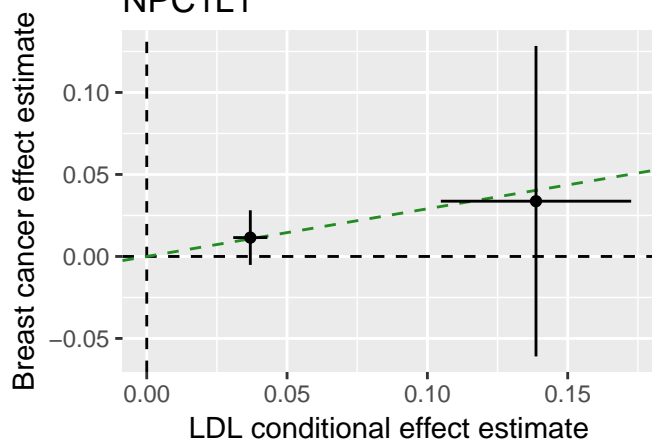

PCSK9

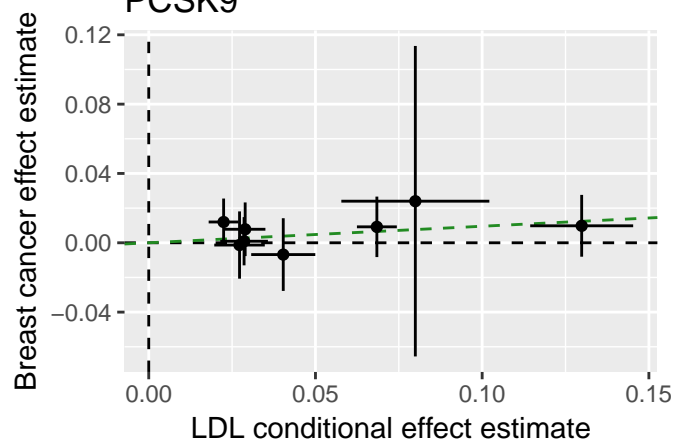

Supplement: S11 Fig — Conditionally independent LDL-associated SNPs at canonical LDL metabolism pathway genes, plotted by their conditional effect estimates on LDL (from MVP) and effect estimates on all BCs. Error bars represent 95% CIs. The dashed green line represents the regression line from fixed-effects IVW MR. BC, breast cancer; CI, confidence interval; IVW, inverse-variance weighted; LDL, low-density lipoprotein; MR, Mendelian randomization; MVP, Million Veteran Program. (PDF) [file pmed.1003302.s013.pdf]

| Gene                 | N SNPs | Odds Ratio (95% CI) | P-value |
|----------------------|--------|---------------------|---------|
| <i>NPC1L1</i>        | 2      | 1.34 (0.92-1.95)    | 0.13    |
| <i>HMGCR</i>         | 2      | 1.23 (1.01-1.51)    | 0.04    |
| <i>LPA</i>           | 4      | 0.95 (0.78-1.15)    | 0.61    |
| <i>APOB</i>          | 7      | 1.08 (0.95-1.23)    | 0.23    |
| <i>LDLR</i>          | 11     | 1.02 (0.91-1.15)    | 0.70    |
| <i>PCSK9</i>         | 8      | 1.10 (0.99-1.23)    | 0.08    |
| <b>Meta-analysis</b> |        | 1.07 (1.01-1.14)    | 0.02    |

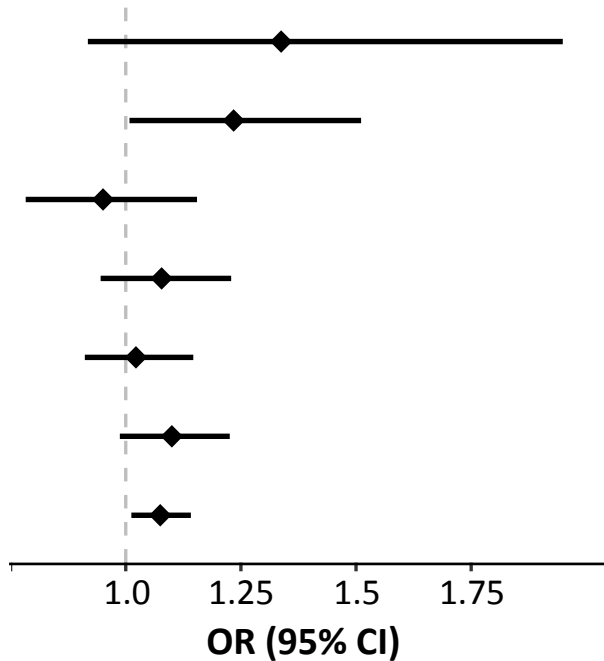

Supplement: S12 Fig — Forest plot of MR results for LDL gene-specific instruments (see S4 Fig) and meta-analysis of effect estimates across genes. Estimates were calculated using a fixed-effects IVW method. BC, breast cancer; CI, confidence interval; IVW, inverse-variance weighted; LDL, low-density lipoprotein; MR, Mendelian randomization; N SNPs, number of genetic instruments included in MR; OR, odds ratio. (PDF) [file pmed.1003302.s014.pdf]

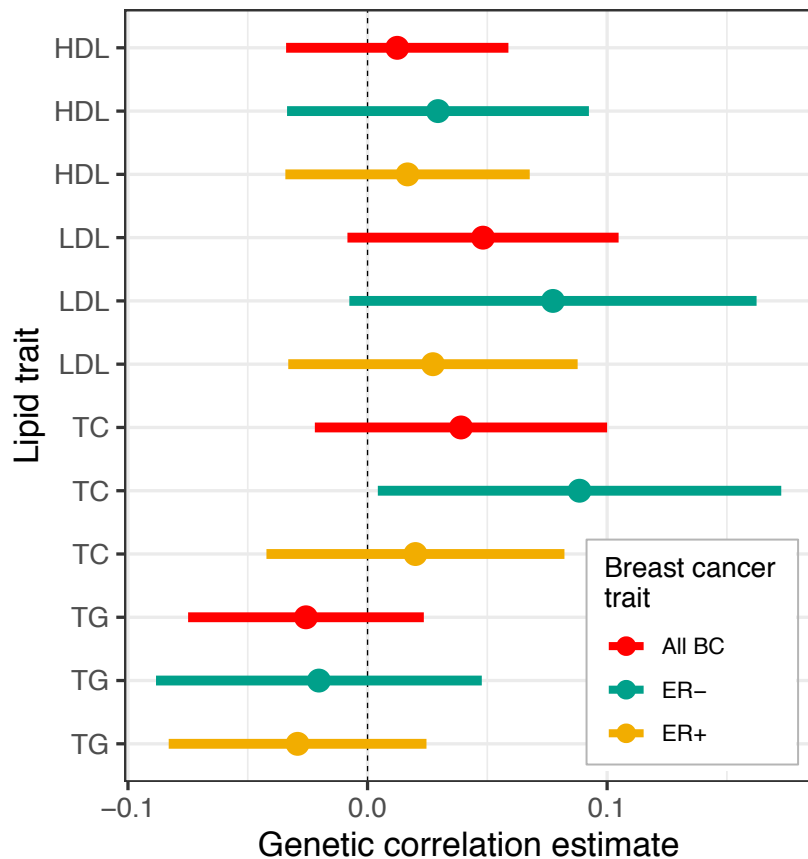

| OR (95% CI)        | P-value |
|--------------------|---------|
| 0.01 (-0.03-0.06)  | 0.59    |
| 0.03 (-0.03-0.09)  | 0.35    |
| 0.02 (-0.03-0.07)  | 0.51    |
| 0.05 (-0.01-0.1)   | 0.09    |
| 0.08 (-0.01-0.16)  | 0.07    |
| 0.03 (-0.03-0.09)  | 0.37    |
| 0.04 (-0.02-0.1)   | 0.20    |
| 0.09 (0-0.17)      | 0.04    |
| 0.02 (-0.04-0.08)  | 0.52    |
| -0.03 (-0.07-0.02) | 0.30    |
| -0.02 (-0.09-0.05) | 0.55    |
| -0.03 (-0.08-0.02) | 0.28    |

Supplement: S13 Fig — Results of LD-score regression testing for genetic correlation between each lipid trait and 3 BC traits: all BC, ER− BCs only, or ER+ BCs only. Error bars represent the 95% CI. Lipid association statistics were from a meta-analysis of GLGC and MVP. BC, breast cancer; CI, confidence interval; ER, estrogen receptor; GLGC, Global Lipids Genetics Consortium; HDL, high-density lipoprotein; LD, linkage disequilibrium; LDL, low-density lipoprotein; MVP, Million Veteran Program; TC; total cholesterol; TG, triglyceride. (PDF) [file pmed.1003302.s015.pdf]
