# Supplementary material for: The relationship between circulating lipids and breast cancer risk: A Mendelian randomization study
Source: PLoS Med. 2020 Sep 11;17(9):e1003302. doi: 10.1371/journal.pmed.1003302 (PMC7485834; doi:10.1371/journal.pmed.1003302)
Supplement: S3 Fig — Scatter plots of genetic instruments included in pruned single-trait MR analyses. Genetic instruments were pruned to pass heterogeneity test. Each plot contains effect estimates from MVP for one of 4 lipid traits (HDL, LDL, TC, TG) on the x-axis and effect estimates for risk of all BCs on the y-axis. Error bars represent the 95% CI, and regression lines represent the slope estimate from one of 3 MR tests: IVW (light blue), Egger regression (dark blue), and weighted median (green). BC, breast cancer; CI, confidence interval; HDL, high-density lipoprotein; IVW, inverse-variance weighted; LDL, low-density lipoprotein; MR, Mendelian randomization; TC, total cholesterol; TG, triglyceride. (PDF) [file pmed.1003302.s005.pdf]

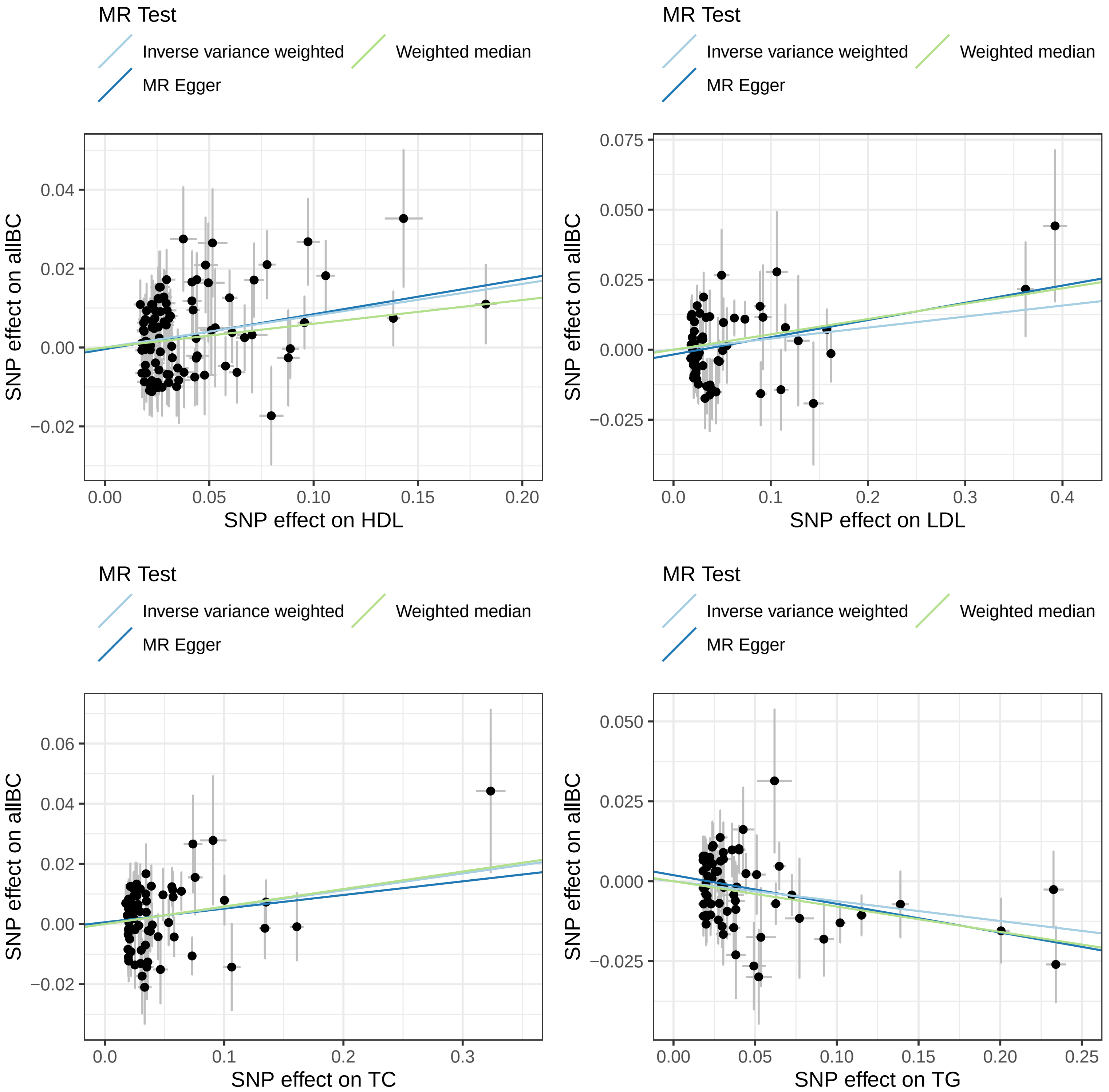

MR Test
Inverse variance weighted
Weighted median
MR Egger
SNP effect on allBC
SNP effect on HDL
0.04
0.02
0.00
−0.02
0.00
0.05
0.10
0.15
0.20
SNP effect on LDL
0.075
0.050
0.025
0.000
−0.025
0.0
0.1
0.2
0.3
0.4
SNP effect on TC
0.06
0.04
0.02
0.00
−0.02
0.0
0.1
0.2
0.3
SNP effect on TG
0.050
0.025
0.000
−0.025
0.00
0.05
0.10
0.15
0.20
0.25
