# Supplementary material for: The relationship between circulating lipids and breast cancer risk: A Mendelian randomization study
Source: PLoS Med. 2020 Sep 11;17(9):e1003302. doi: 10.1371/journal.pmed.1003302 (PMC7485834; doi:10.1371/journal.pmed.1003302)
Supplement: S1 Text — Included are details about the GWASs utilized, heterogeneity analysis for single-trait MR, instrument strength and validity assessment for multivariable MR, and a list of investigators associated with the VA MVP (banner author). GWAS, genome-wide association study; MR, Mendelian randomization; MVP, Million Veteran Program; VA, Veterans Affairs. (DOCX) [file pmed.1003302.s031.docx]

# Supplementary Text

GWAS details

Million Veteran Program (MVP) lipid summary statistics were estimated with European-ancestry individuals with the following sample sizes: 210,967 (HDL), 215,196 (LDL), 215,551 (total cholesterol), 211,491 (TG) [1]. Lipids GWAS summary statistics from the Global Lipids Genetics Consortium (GLGC) [2] were downloaded from <http://csg.sph.umich.edu/abecasis/public/lipids2013/> on March 3rd, 2017; this study included up to 188,577 European-ancestry genotyped individuals.

Genome-wide association study (GWAS) summary statistics for breast cancer [3] from the Breast Cancer Association Consortium (BCAC) were downloaded from <http://bcac.ccge.medschl.cam.ac.uk/bcacdata/> on October 27th, 2017. An assessment of heterogeneity in the BCAC data is available in Supplementary table 28 in this manuscript. This study performed a GWAS meta-analysis of a total of 122,977 breast cancer cases and 105,974 controls, and also breast cancer subtype meta-analyses with 69,501 cases (ER+) or 21,468 cases (ER-). In a multivariable MR experiment, we used summary statistics from three independent subsets of the BCAC consortium dataset: “Oncoarray”, 61,282 female cases with breast cancer and 45,494 female controls; “iCOGS”, 46,785 cases and 42,892 controls; and “GWAS”, 14,910 cases and 17,588 controls from 11 GWAS. Breast cancer genome-wide association analyses were supported by the Government of Canada through Genome Canada and the Canadian Institutes of Health Research, the ‘Ministère de l’Économie, de la Science et de l’Innovation du Québec’ through Genome Québec and grant PSR-SIIRI-701, The National Institutes of Health (U19 CA148065, X01HG007492), Cancer Research UK (C1287/A10118, C1287/A16563, C1287/A10710) and The European Union (HEALTH-F2-2009-223175 and H2020 633784 and 634935). All studies and funders are listed in [3]. There is no known cohort overlap between the MVP and BCAC datasets. BMI summary statistics used in our multivariable MR (from Yengo et al) are available at https://portals.broadinstitute.org/collaboration/giant/index.php/GIANT_consortium_data_files#2018_GIANT_and_UK_BioBank_Meta-analysis. Age of menarche summary statistics (from Day et al) are available at https://www.reprogen.org/data_download.html.

This study data generated by the UK10K Consortium, derived from samples from ALSPAC and TWINSUK, as an LD reference panel for pruning genetic instruments. A full list of the investigators who contributed to the generation of the data is available from [www.UK10K.org](http://www.uk10k.org/). Funding for UK10K was provided by the Wellcome Trust under award WT091310.

Heterogeneity analyses for single trait MR

Cochran’s Q revealed significant heterogeneity in the genetic instruments’ ratios of effects on outcome and exposure in our initial single trait inverse-variance-weighted MR analyses (**Supplementary Table 5**) [4,5]. This heterogeneity suggests pleiotropic effects in the outlier SNPs. We constructed a second genetic instrument subjected to the following pruning procedure to ensure the genetic instruments for each lipid trait met the assumption of no significant heterogeneity of effects. Starting with a set of genetic instruments of all unlinked SNPs (r^2^ < 0.001) reaching genome-wide significance (P < 5 x 10^-8^) for association with each lipid trait, we performed Cochran’s Q test for heterogeneity [4], and removed SNPs with the largest contributions to Q until the null hypothesis of Cochran’s Q test was no longer rejected (P > 0.05). Using Cochran’s Q with inverse-variance weights makes the NOME (no measurement error assumption), which can become problematic in the presence of weak genetic instruments [5]. Due to the large GWAS sample size and significant heritability of the lipid traits used here, strong measurement error or weak instruments are not significant concerns and as a result Cochran’s Q is an appropriate test of heterogeneity. By removing potentially pleiotropic variants from our single-trait and multivariable MR analyses (see below), we better satisfy the assumptions of causal inference via MR [6]. Importantly, we note that the estimates from our MR analyses before and after pruning for instrument heterogeneity are in strong agreement.

Multivariable MR tests of instrument strength and validity

We performed two heterogeneity tests with our multivariable MR genetic instruments [7], which are both modified Cochran’s test of heterogeneity, to evaluate instrumental variable strength (Q_X1_) or validity (Q_A_) in a two-sample (or more) summary data setting. We found that our instrumental variables could predict each exposure trait when conditioning on the other exposures (Q_X1_), but that there was significant heterogeneity suggesting invalid instruments when all significant genetic instruments were included (Q_A_). As in single trait MR, heterogeneity of genetic instrument effects in multivariable MR can indicate pleiotropy, or invalid instruments. To account for this, we further pruned our genetic instruments used for our multivariable analysis, using a stepwise post-hoc procedure to remove genetic instruments that contributed the most to Q_A_ until the statistic was below the critical value (α = 0.05). To calculate Q_X1_ and Q_A_, the covariance between the genetic instruments’ effects on each exposure must be estimated from individual-level data or assumed to be zero [7]. As we did not have access to individual-level data, we assumed this covariance term was zero. To test the effect of this assumption, we also performed multivariable MR using non-overlapping samples (using MVP paired with GLGC) for each of the lipid exposure traits (Supplementary Figure 7, Supplementary Table 11), thus fixing covariance at zero.

# Works Cited

1. Klarin D, Damrauer SM, Cho K, Sun Y V., Teslovich TM, Honerlaw J, et al. Genetics of blood lipids among ~300,000 multi-ethnic participants of the Million Veteran Program. Nat Genet. 2018;50: 1514–1523. doi:10.1038/s41588-018-0222-9

2. Global Lipids Genetics Consortium, Willer CJ, Schmidt EM, Sengupta S, Peloso GM, Gustafsson S, Kanoni S, et al. Discovery and Refinement of Loci Associated with Lipid Levels. Nat Genet. 2013;45: 1274–1283. doi:10.1038/ng.2797.Discovery

3. Michailidou K, Lindström S, Dennis J, Beesley J, Hui S, Kar S, et al. Association analysis identifies 65 new breast cancer risk loci. Nature. 2017;551: 92–94. doi:10.1038/nature24284

4. Bowden J, Hemani G, Davey Smith G. Invited Commentary: Detecting Individual and Global Horizontal Pleiotropy in Mendelian Randomization—A Job for the Humble Heterogeneity Statistic? Am J Epidemiol. 2018;187: 2681–2685. doi:10.1093/aje/kwy185

5. Bowden J, Del Greco M F, Minelli C, Zhao Q, Lawlor DA, Sheehan NA, et al. Improving the accuracy of two-sample summary-data Mendelian randomization: Moving beyond the NOME assumption. Int J Epidemiol. 2019;48: 728–742. doi:10.1093/ije/dyy258

6. Burgess S, Davey Smith G, Davies NM, Dudbridge F, Gill D, Glymour MM, et al. Guidelines for performing Mendelian randomization investigations. Wellcome Open Res. 2019;4. doi:10.12688/wellcomeopenres.15555.1

7. Sanderson E, Davey Smith G, Windmeijer F, Bowden J. An examination of multivariable Mendelian randomization in the single-sample and two-sample summary data settings. Int J Epidemiol. 2019;48: 713–727. doi:10.1093/ije/dyy262

# List of VA Million Veteran Program Contributors

**MVP Executive Committee**

- Co-Chair: J. Michael Gaziano, M.D., M.P.H.
- Co-Chair: Rachel Ramoni, D.M.D., Sc.D.
- Jim Breeling, M.D. (ex-officio)
- Kyong-Mi Chang, M.D.
- Grant Huang, Ph.D.
- Sumitra Muralidhar, Ph.D.
- Christopher J. O’Donnell, M.D., M.P.H.
- Philip S. Tsao, Ph.D.

**MVP Program Office**

- Sumitra Muralidhar, Ph.D.
- Jennifer Moser, Ph.D.

**MVP Recruitment/Enrollment**

- Recruitment/Enrollment Director/Deputy Director, Boston – Stacey B. Whitbourne, Ph.D.; Jessica V. Brewer, M.P.H.
- MVP Coordinating Centers
  - Clinical Epidemiology Research Center (CERC), West Haven – John Concato, M.D., M.P.H.
  - Cooperative Studies Program Clinical Research Pharmacy Coordinating Center, Albuquerque - Stuart Warren, J.D., Pharm D.; Dean P. Argyres, M.S.
  - Genomics Coordinating Center, Palo Alto – Philip S. Tsao, Ph.D.
  - Massachusetts Veterans Epidemiology Research Information Center (MAVERIC), Boston - J. Michael Gaziano, M.D., M.P.H.
  - MVP Information Center, Canandaigua – Brady Stephens, M.S.
- Core Biorepository, Boston – Mary T. Brophy M.D., M.P.H.; Donald E. Humphries, Ph.D.
- MVP Informatics, Boston – Nhan Do, M.D.; Shahpoor Shayan
- Data Operations/Analytics, Boston – Xuan-Mai T. Nguyen, Ph.D.

**MVP Science**

- Genomics - Christopher J. O’Donnell, M.D., M.P.H.; Saiju Pyarajan Ph.D.; Philip S. Tsao, Ph.D.
- Phenomics - Kelly Cho, M.P.H, Ph.D.
- Data and Computational Sciences – Saiju Pyarajan, Ph.D.
- Statistical Genetics – Elizabeth Hauser, Ph.D.; Yan Sun, Ph.D.; Hongyu Zhao, Ph.D.

**MVP Local Site Investigators**

- Atlanta VA Medical Center (Peter Wilson)

1670 Clairmont Rd, Decatur, GA 30033

- Bay Pines VA Healthcare System (Rachel McArdle)

10,000 Bay Pines Blvd Bay Pines FL 33744

- Birmingham VA Medical Center (Louis Dellitalia)

700 S. 19th Street Birmingham AL 35233

- Cincinnati VA Medical Center (John Harley)

3200 Vine Street, Cincinnati, OH 45220

- Clement J. Zablocki VA Medical Center (Jeffrey Whittle)

5000 West National Avenue, Milwaukee, WI 53295

- Durham VA Medical Center (Jean Beckham)

508 Fulton Street Durham, NC 27705

- Edith Nourse Rogers Memorial Veterans Hospital (John Wells)

200 Springs Road, Bedford, MA 01730

- Edward Hines, Jr. VA Medical Center (Salvador Gutierrez)

5000 South 5th Avenue, Hines, IL 60141

- Fayetteville VA Medical Center (Gretchen Gibson)

1100 N College Ave, Fayetteville, AR 72703

- VA Health Care Upstate New York (Laurence Kaminsky)

113 Holland Avenue Albany NY 12208

- New Mexico VA Health Care System (Gerardo Villareal)

1501 San Pedro Drive, S.E.Albuquerque, NM 87108

- VA Boston Healthcare System (Scott Kinlay)

150 S. Huntington Avenue, Boston, MA 02130

- VA Western New York Healthcare System (Junzhe Xu)

3495 Bailey Avenue Buffalo, NY 14215-1199

- Ralph H. Johnson VA Medical Center (Mark Hamner)

109 Bee Street, Mental Health Research, Charleston, SC 29401

- Wm. Jennings Bryan Dorn VA Medical Center (Kathlyn Sue Haddock)

6439 Garners Ferry Road, Columbia, SC 29209

- VA North Texas Health Care System (Sujata Bhushan)

4500 S. LANCASTER ROAD, DALLAS, TX 75216

- Hampton VA Medical Center (Pran Iruvanti)

100 Emancipation Drive, Hampton, VA 23667

- Hunter Holmes McGuire VA Medical Center (Michael Godschalk)

1201 Broad Rock Blvd., Richmond, VA 23249

- Iowa City VA Health Care System (Zuhair Ballas)

601 Highway 6 West, Iowa City, IA 52246-2208

- Jack C. Montgomery VA Medical Center (Malcolm Buford)

1011 Honor Heights Dr., Muskogee, OK 74401

- James A. Haley Veterans’ Hospital (Stephen Mastorides)

13000 Bruce B. Downs Blvd., Tampa, FL 33612

- Louisville VA Medical Center (Jon Klein)

800 Zorn Avenue, Louisville, KY 40206

- Manchester VA Medical Center (Nora Ratcliffe)

718 Smyth Road, Manchester, NH 03104

- Miami VA Health Care System (Hermes Florez)

1201 NW 16th Street, 11 GRC, Miami FL 33125

- Michael E. DeBakey VA Medical Center (Alan Swann)

2002 Holcombe Blvd. Houston TX 77030

- Minneapolis VA Health Care System (Maureen Murdoch)

One Veterans Drive Minneapolis MN 55417

- N. FL/S. GA Veterans Health System (Peruvemba Sriram)

1601 SW Archer Road, Gainesville, FL 32608

- Northport VA Medical Center (Shing Shing Yeh)

79 Middleville Road, Northport, NY 11768

- Overton Brooks VA Medical Center (Ronald Washburn)

510 East Stoner Ave, Shreveport, LA 71101

- Philadelphia VA Medical Center (Darshana Jhala)

3900 Woodland Avenue, Philadelphia, PA 19104

- Phoenix VA Health Care System (Samuel Aguayo)

650 E. Indian School Road, Phoenix, AZ 85012

- Portland VA Medical Center (David Cohen)

3710 SW U.S. Veterans Hospital Road, Portland, OR 97239

- Providence VA Medical Center (Satish Sharma)

830 Chalkstone Avenue, Providence, RI 02908

- Richard Roudebush VA Medical Center (John Callaghan)

1481 West 10th Street, Indianapolis, IN 46202

- Salem VA Medical Center (Kris Ann Oursler)

1970 Roanoke Blvd.,Salem, VA 24153

- San Francisco VA Health Care System (Mary Whooley)

4150 Clement Street, San Francisco, CA 94121

- South Texas Veterans Health Care System (Sunil Ahuja)

7400 Merton Minter Boulevard, San Antonio, TX 78229

- Southeast Louisiana Veterans Health Care System (Amparo Gutierrez)

2400 Canal Street, New Orleans, LA 70119

- Southern Arizona VA Health Care System (Ronald Schifman)

3601 S 6th Ave, Tucson, AZ 85723

- Sioux Falls VA Health Care System (Jennifer Greco)

2501 W 22nd St, Sioux Falls, SD 57105

- St. Louis VA Health Care System (Michael Rauchman)

915 North Grand Blvd., St. Louis, MO 63106

- Syracuse VA Medical Center (Richard Servatius)

800 Irving Avenue, Syracuse, NY 13210

- VA Eastern Kansas Health Care System (Mary Oehlert)

4101 S 4th Street Trafficway, Leavenworth, KS 66048

- VA Greater Los Angeles Health Care System (Agnes Wallbom)

11301 Wilshire Blvd Los Angeles, CA 90073

- VA Loma Linda Healthcare System (Ronald Fernando)

11201 Benton Street, Loma Linda, CA 92357

- VA Long Beach Healthcare System (Timothy Morgan)

5901 East 7th Street Long Beach CA 90822

- VA Maine Healthcare System (Todd Stapley)

1 VA Center, Augusta, ME 04330

- VA New York Harbor Healthcare System (Scott Sherman)

423 East 23rd Street New York, NY 10010

- VA Pacific Islands Health Care System (Gwenevere Anderson)

459 Patterson Rd, Honolulu, HI 96819

- VA Palo Alto Health Care System (Philip Tsao)

3801 Miranda Avenue Palo Alto, CA 94304-1290

- VA Pittsburgh Health Care System (Elif Sonel)

University Drive, Pittsburgh, PA 15240

- VA Puget Sound Health Care System (Edward Boyko)

1660 S. Columbian Way Seattle, WA 98108-1597

- VA Salt Lake City Health Care System (Laurence Meyer)

500 Foothill Drive Salt Lake City, UT 84148

- VA San Diego Healthcare System (Samir Gupta)

3350 La Jolla Village Drive, San Diego, CA 92161

- VA Southern Nevada Healthcare System (Joseph Fayad)

6900 North Pecos Road, North Las Vegas, NV 89086

- VA Tennessee Valley Healthcare System (Adriana Hung)

1310 24th Ave. South Nashville, TN 37212

- Washington DC VA Medical Center (Jack Lichy)

50 Irving St, Washington, D. C. 20422

- W.G. (Bill) Hefner VA Medical Center (Robin Hurley)

1601 Brenner Ave, Salisbury, NC 28144

- White River Junction VA Medical Center (Brooks Robey)

163 Veterans Drive, White River Junction, VT 05009

- William S. Middleton Memorial Veterans Hospital (Robert Striker)
- 2500 Overlook Terrace, Madison, WI 53705
