# Supplementary material for: The relationship between circulating lipids and breast cancer risk: A Mendelian randomization study
Source: PLoS Med. 2020 Sep 11;17(9):e1003302. doi: 10.1371/journal.pmed.1003302 (PMC7485834; doi:10.1371/journal.pmed.1003302)
Supplement: Attachment — Submitted filename: Response_to_reviewers_2.pdf [file pmed.1003302.s032.pdf]

Requests from the editors:

1. Data Availability: Thank you for pointing out that your data are freely available. In your data availability statement, please state the location of data both within the paper, and also provide the links (as you have done in the supporting information section).

We have added the URLs and accession numbers of all GWAS data sets used to the Data Availability Statement. In addition, we have added that the summary statistics for the instrumental variables we used are available in Supplementary Tables 1, 2 and 3.

2. Did your study have a prospective protocol or analysis plan? Please state this (either way) early in the Methods section.

We did not have a prospective protocol. We have added a statement to the methods corresponding to the Analysis Plan that we followed (lines 197- 206).

a) If a prospective analysis plan (from your funding proposal, IRB or other ethics committee submission, study protocol, or other planning document written before analyzing the data) was used in designing the study, please include the relevant prospectively written document with your revised manuscript as a Supporting Information file to be published alongside your study, and cite it in the Methods section. A legend for this file should be included at the end of your manuscript.

N/A

b) If no such document exists, please make sure that the Methods section transparently describes when analyses were planned, and when/why any data-driven changes to analyses took place.

We have added an "Analysis Plan" section to the Methods (lines 197- 206).

c) In either case, changes in the analysis-- including those made in response to peer review comments-- should be identified as such in the Methods section of the paper, with rationale.

Understood.

3. Title: Please revise your title to: "The relationship between circulating lipids and breast cancer risk: a Mendelian randomization study" or similar.

We have changed the title to the editors' suggestion.

4. Abstract: Line 10: The word "randomization" is missing at the end of the last sentence of the background.

We have added in this word.

5. Abstract: Please define abbreviations for BCAC, SD, OR, MR at their first use. Please also give some brief context for "ABO locus", to indicate relevance for blood group, etc.

We have added in the definitions of these abbreviations (lines 132, and 77 for BCAC and MR), spelt out SD and OR when used instead of abbreviation, and added context for the ABO locus to our results section.

6. Abstract: In the last sentence of the Abstract Methods and Findings section, please describe the main limitation(s) of the study's methodology.

We have added a sentence discussing the main limitations--namely confounding due to pleiotropy and population stratification.

7. Abstract: Line 27: Please change "find" to "found".

We have made this change.

8. Abstract: Conclusions: Please modify the first sentence of the conclusion to: "Genetically elevated plasma HDL levels appear to be associated with increased breast cancer risk" or similar. The phrase "In this study, we observed ..." may be useful.

We have modified this sentence as suggested.

9. At this stage, we ask that you include a short, non-technical Author Summary of your research to make findings accessible to a wide audience that includes both scientists and non-scientists. The Author Summary should immediately follow the Abstract in your revised manuscript. This text is subject to editorial change and should be distinct from the scientific abstract. Please see our author guidelines for more information: https://journals.plos.org/plosmedicine/s/revising-your-manuscript#loc-author-summary

We have added an Author summary.

10. Introduction: Line 56: Please define the abbreviation for GWAS at first use.

We have now spelt out the GWAS acronym upon first use (line 83).

11. Introduction: Line 58: Please define the abbreviation for "ER"

We have now added that this stands for estrogen receptor (line 85).

12. Introduction: Line 82-83: Please remove the description of the findings from the introduction.

We have removed the description of our findings from the intro.

13. Methods: Please include a statement to generally describe that institutional ethical approval and consent were obtained for those participating in the cohorts that provided the summary/ GWAS data.

We have added this sentence to the methods section (lines 134-135).

14. Methods: Line 101: Should "plink" be capitalized?

Yes, we have replaced plink with "PLINK" (line 140)

15. Methods: Line 117-119: Please make sure that the referencing of these studies/criteria follow Vancouver style: https://journals.plos.org/plosmedicine/s/submission-guidelines#loc-references

We have fixed our citation style.

16. Methods: Line 139: please define abbreviation for LD.

We have now added what LD stands for (line 86).

17. Results: Line 151: Thank you for spelling out these abbreviations- however, please spell them out where they are first used earlier in the manuscript.

We have now said what they stand for when first used (lines 80-81).

18. Results: Line 248-251: For clarity, we suggest that you first mention only the significant interactions (and the method that produced them) and then list the methods/interactions that fall short of significant second. Please explicitly clarify where significance indicates statistical significance, and please clarify what is meant by "nominal association" - does this indicate statistical significance? If so, please state that.

We have now restructured this paragraph (now lines 320-331) accordingly.

19. Results: Throughout the results section, where positive or negative relationships are noted, please clarify whether relationships are statistically significant, for example at Line 230: "We observed a positive relationship…" it seems such relationships reach statistical significance, while relationships described at line 248 do not. We suggest revising the sentence: "However, most P-values were not significant; the only nominal association ($P < 0.05$) was with total cholesterol and ER-negative breast cancer ($P=0.04$)." to: "However, most genetic correlation estimates between breast cancer and lipid traits were not statistically significant; the only nominal statistically significant association ($P < 0.05$) was with total cholesterol and ER-negative breast cancer ($P=0.04$)." or similar.

We now specify whether each relationship in the results section is significant or not.

20. Figure 1: Some symbols in Figure 1 do not appear to be displaying properly, please check the formatting. Please define all abbreviations in the figure legend (i.e. for MR, OR, VW, HDL, LDL, TG).

We now define all abbreviations in the legend. We have regenerated our figures, and they look good in PACE, so hopefully the symbols display properly on all computers now.

21. Figure 2: Please define abbreviations "N SNPs" and "OR" in the figure legend. In the right-side panel, please describe the dotted line and bars.

Figure legend has been expanded to include these definitions.

22. Discussion: Line 308: Please define abbreviation for "IVS".

We have now simply spell out "instrumental variables" on line 408.

23. Discussion: Line 323: Please edit the sentence to "These findings support a causal relationship between increased HDL cholesterol and increased breast cancer risk…" or similar.

We have revised this sentence to the following:
"These findings support a causal relationship between increased HDL cholesterol and increased breast cancer risk, and this hypothesis warrants further exploration."

24. References: For in-text citations, please use square brackets, like this [1].

We have changed our citations to square brackets.

25. Supporting Information: Please provide titles for supplementary figures and tables, and please ensure that all abbreviations present in the figures and tables are defined in the figure legends.

We have added titles for all supplementary figures and tables, and defined all abbreviations.

26. Supporting Information: For consistency, please use the "Vancouver" style for reference formatting, and see our website for other reference guidelines:
https://journals.plos.org/plosmedicine/s/submission-guidelines#loc-references

We have switched to Vancouver style for our SI and main text.

Comments from the reviewers:

Reviewer #1: PMEDICINE-D-19-03886.

The authors conduct a nice, thorough Mendelian Randomization analysis of the relationship between blood lipids and breast cancer risk. Similar work has been done previously (Nowak and Ärnlöv, Nat Comm 2018; Qi and Chatterjee Nat Comm 2019). Nonetheless, the work presented here constitutes a more comprehensive MR analysis than those papers, but the authors should cite the work by Qi and Chatterjee. I have a few questions for clarification

We thank this reviewer for their support and suggestions for our paper. We regret not having added that work to our citations in our initial submission; we have added a citation and discussion of the Qi & Chatterjee study to our Introduction (line 92).

1. It is not clear to me why the authors sometimes uses lipids summary statistics from MVP, sometimes from GLGC and sometimes from the meta-analysis between the two.

We focus on MVP, because an MR analysis on breast cancer using this dataset is independent of prior studies which used GLGC data. We choose to present secondary analyses of the MVP/GLGC meta-analysis and just GLGC to gain the highest power analysis and to show that our effects are not specific to one lipid GWAS. If we did see substantial heterogeneity between the MR effects estimated using just the MVP or GLGC GWAS, this could indicate that our results were being confounded by effects such as population stratification.

2. There is some evidence of sex difference in SNP-lipids associations (e.g. Asselbergs, AJHG 2012). If possible, it would be interesting to see MR analysis using SNPs associated with lipids specifically in women, although this might not be possible if the authors do not have access to the raw GWAS and lipids data for such analyses.

This is indeed an interesting suggestion. Unfortunately, we do not have access to a sex-stratified lipid GWAS, so this would have to be something to consider for future work.

3. It is not clear to me why the authors present data from iCOGS, OncoArray and GWAS separately? Unless there are explicit reasons for this, I suggest only using the meta-analysis results for breast cancer only.

We agree; the main results present in the text correspond to the combined data set. The sub-strata analyses we performed in our multivariate MR experiment with the three independent subsets of the BCAC meta-analysis was simply as a sensitivity and robustness test to examine potential heterogeneity of the effects of the exposures on breast cancer risk. This experiment confirmed that the meta-analysis results are consistent across cohort subsets. We leave this in the supplement for interested readers.

4. The authors pruned pleiotropic variants and reran the analyses similar to the Nowak paper (top page 10). Even though they note that the confidence intervals increased, it is also worth noting that the effect estimates changed quite a bit as well, so the null association is not only due to the increased confidence intervals. In particular, for TG the confidence intervals barely overlap.

We agree that the change in effect estimates is worth noting, and have edited this sentence in the manuscript to the following:

"After this pruning, we did not find a significant relationship with LDL, HDL, or TG; and we note that this pruning procedure resulted in considerably larger confidence intervals spanning OR=1 for all traits, with reversed direction of effect estimates for LDL and TG (**Supplementary Table 9**)."

5. As the authors pointed out, one of the things that distinguish this work from Nowak and Ärnlöv is the multivariable approach presented here. Another strength with this paper is the assessment of ER+ and ER- breast cancer. I think that the paper would gain from a more comprehensive analysis of ER+ and ER- breast cancer specifically, using some of the multivariable approaches described here.

We thank the reviewer for their suggestion, which strengthens our results. We performed an additional multivariable MR analysis using the genetic associations for just ER+ or ER- breast cancer as the outcome (now Supplementary Figure 9, pasted below). Consistent with our single-trait MR results, the breast cancer subtypes had consistent relationships with each exposure trait.

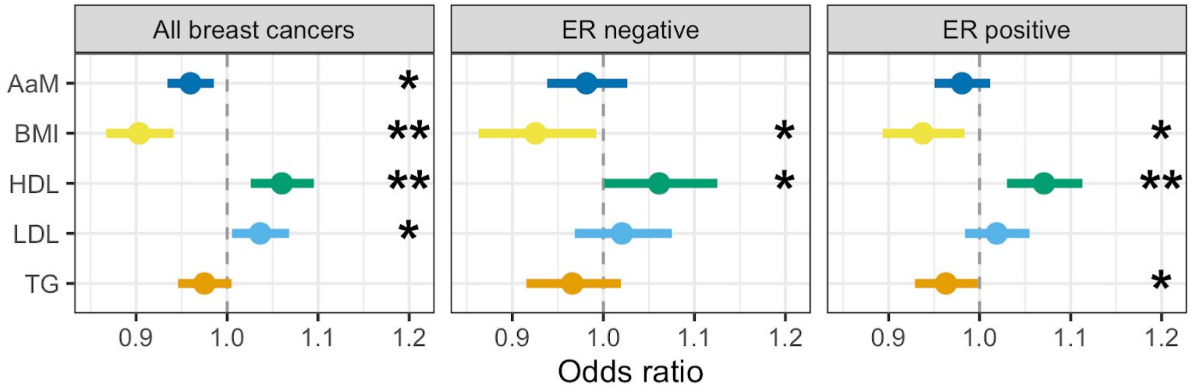

6. Are the results from rho-HESS (Suppl Table 13) genetic covariances or genetic correlations? They seem small for being correlations. For the uninformed, a quick explanation about the differences in the overall (not local) genetic correlations as estimated from rho-HESS and LDSC would be helpful and help interpret the differences in results. Related to that, it would be interesting to expand on the discussion on the local genetic correlation results. Is the main conclusion from those analyses that ABO is casually associated with both blood lipids and breast cancer risk?

We thank this reviewer for catching a significant issue with our column headers in Supplementary Table 13 (now Supplementary Table 14). The genetic covariances were correctly labeled, however due to a

column header mix-up, the z-score column actually displayed the genetic correlations, while the p-value column displayed the genetic correlation standard errors. We have now fixed this table so it displays both the genetic correlations, covariances and the corresponding standard errors. We note that when using the correct p-values, only TC and LDL have a significant genetic correlation with BC.

One main difference between ρ-Hess and LDSC is that the ρ-Hess method uses a truncated singular-value decomposition regularization step to remove noise in external reference LD. They note that their estimator provides lower standard errors as compared to cross-trait LDSC and is likely due to the truncated-SVD regularization procedure. The authors of that work demonstrated in simulation that their approach performs better with superior accuracy, and they conjecture that the regularization procedure is the main reason for this (reading carefully, this has to do with reducing the degrees of freedom required when estimating the covariances given the LD matrix). The reduction in the standard error of covariance estimates obviously directly relates to increased power for discovery of true correlations, which may be why we make a positive discovery here (for LDL and all-cause breast cancer). In addition, LDSC assumes a random effect for the SNP effect sizes, whereas ρ-Hess assumes a fixed effect model. In specific scenarios, the random effect sizes could also increase the estimated standard error relative to a fixed-effect model. We have added these points (without going into too many mathematical details for the clinical audience) to the discussion.

"The discrepancy between LDSC and ρ-Hess may be due to a difference in the statistical power of these methods, which has been previously reported [38]."

We cannot make a claim for causality at the ABO locus. The best we can say is that there is a local correlation between LDL and Breast-cancer risk increasing haplotypes in the region. We have added a short bit of discussion accordingly:

"Furthermore, we find evidence of genome-wide genetic correlation between some lipid traits and BC, and local genetic correlation at the ABO locus. Although some studies have found an association between blood group and breast cancer risk [47], haplotype patterns indicate that ABO gene expression, not blood group, may be the causal mechanism [39]. However, because of the pleiotropic nature of the ABO locus, it is unclear whether the breast cancer association is caused by the lipid associations [41]."

7. Minor point: Refs no. 5 and 7 seem to be the same.

Thanks for catching this mistake. Our reference numbers now correctly map to the cited papers.

Reviewer #2: This is a comprehensive Mendelian randomization study of lipids and breast cancer risk. Though I am not familiar with the more novel methods used in the study, it is clear that the authors apply both conventional and state-of-the-art methods on well-known large GWAS data to investigate the topic in depth. The study is well-written, and the authors generally use balanced language in relation to their methods and findings. I have few specific comments:

We thank this reviewer for their positive feedback and comments.

Introduction

1. Reverse causation has been frequently debated to be involved in the association between cholesterol and risk of several forms of cancer. I suggest to mention the potential for reverse causation also in breast cancer if this is of particular relevance also for breast cancer.

We agree, and we believe that the MR approach can address issues of reverse causation which could be problematic in observational studies, in which lipid levels are measured in individuals with breast cancer, and we mention this in the revised discussion. As breast cancer is relatively rare, the majority of patients analyzed in the lipids genetic study are unlikely to contain individuals who have experienced breast cancer, so the potential for biases there is low.

We have added this sentence to the discussion:
"Our analyses help clarify these mixed results, and infer a direction of effect, which is not possible in observational studies due to potential reverse causation"

2. Reference 6 is about survival amongst cancer cases and not about cancer risk, as referred to in the introduction and which the present study is about. The reference also lacks authors in the reference list.

We agree, and have updated our wording and the authors listed in the reference.

3. Reference 7 is not about statins, but is a meta-analysis of lipids and breast cancer risk. Reference 5 and 7 are the same.

We have corrected our citations, and reference 7 is now the correct study: "Statin use and mortality in cancer patients…".

4. In reference 9, an equally suggestive positive association between HDL and breast cancer was shown as the suggestive negative association between TG and breast cancer. Also, for TG, the authors should report the association in ref 9 to be inverse/negative.

We agree that the slightly less conclusive association reported for HDL is relevant to our paper, so we have added mention of it: "In a small sample of 1,187 breast cancer cases, Orho-Melander *et al*. used multivariable MR to find suggestive evidence of a relationship between both triglycerides and HDL (high density lipoprotein) cholesterol and breast cancer, but no association between LDL (low density lipoprotein cholesterol) cholesterol and cancer [9]."

Reference 9 and 38 are the same. Reference 38 referred to as the study by Nowak et al (first sentence page 15) is wrong.

Thanks to the reviewer for catching this, we have adjusted these to what we think are the correct citations for the points we aim to make. We have edited the sentence ("Although Nowak et al. previously…") to

only cite the Nowak et al. study, and removed the duplicate citation for Orho-Melander from our bibliography.

5. There are several strong risk factors of breast cancer in addition to BMI (BMI not even being amongst the most important ones) and age at menarche, so including particularly these in an MR analysis is not the "ideal approach", the authors should remove or reformulate the sentence on page 4, lines 69-71.

We apologize if this was confusing. Our initial text was not intended to say that the approach was the best approach, but merely that it was an improvement over previous work and approaches that did not correct for these confounders explicitly. We have modified wording in this section, including removal of the word ideal, to clarify our meaning (lines 99-101).

6. Please remove the plentiful results reported from the last paragraph of the introduction, as they are not essential to understand the aims.

We agree, and have shortened this section to only include an overview of the approaches used, and not the results.

Methods
1. Page 6, lines 107-108. Please revise the sentence if the lipid, BMI and age at menarche genetic associations were estimated in different cohorts to those of breast cancer genetic associations, i.e. the important part being no overlap with breast cancer cohorts.

We have revised this sentence as follows:

For all analyses, we used a two-sample MR framework, with exposure(s) (lipids, BMI, age at menarche) and outcome (breast cancer) genetic associations from separate cohorts.

2. Include a reference for the multivariable MR analysis performed.

We have added the following two citations to our Methods section (the first introduces two-sample MVMR with summary statistics, the second describes the IVW method we used):

33. Burgess S, Thompson SG. Multivariable Mendelian randomization: The use of pleiotropic genetic variants to estimate causal effects. Am J Epidemiol. 2015;181: 251–260. doi:10.1093/aje/kwu283

34. Burgess S, Dudbridge F, Thompson SG. Re: "Multivariable Mendelian randomization: The use of pleiotropic genetic variants to estimate causal effects." Am J Epidemiol. 2015;181: 290–291. doi:10.1093/aje/kwv017

Reviewer #3: This study uses genetic instruments defined in the Million Veterans Project, to assess the potential role of HDL and LDL in breast cancer risk data from the BCAC consortium.

This study ignores literature published during 2019, when 2 other papers on Mendelian randomisation provide essentially the same results. One of them, by Beeghly-Fadiel et al. on behalf of the BCAC group, had access to individual level data to stratify the analyses not only by ER status, but also for menopausal status, age, and BMI. In that study, only the genetic instrument for HDL was associated with BC. The second study, by Qi and Chatterjee also found the increased risk restricted to HDL, not to LDL or TG. The authors should revise their manuscript in light of these of the studies and explain the reason of their different findings.

We thank the reviewer for pointing us to these two pieces of work. We note the study of Beeghly-Fadiel was recently published during the course of our manuscript's review at this journal (PLoS Medicine), evidenced by the pre-print of our manuscript we posted in late October prior to submitting here (https://www.biorxiv.org/content/10.1101/794594v3). As a result, we were not aware of this work during this initial review, and thus did not comment on it.

That now said, there are differences between both studies, with complementary and detailed types of analyses presented in both. However, we believe that our work stands separately and the collection of both pieces of work provides a strong case for HDL as a potential risk factor.

We regret that we did not include Qi and Chatterjee in our summary of the literature. As reviewer #1 pointed out, our work also stands in complement to Qi and Chatterjee et al; their main focus is on methodology (reporting a nominal finding for HDL without considering confounding factors explicitly), whereas our main focus is to understand the role of HDL/LDL by controlling for several known factors explicitly, and by jointly considering lipids traits as a collection in much more exhaustive multivariable MR studies.

On these points, we have added a discussion of the similarities and differences between the two studies to our Discussion.

The adjustment for BMI and age at menarche, using genetic instruments is an interesting approach when no observed data is accessible, but it should be commented what are the R2 of these instruments? if low, residual confounding may be high. The study bye the BCAC group analysed these associations among controls and only found consistent associations with age. Similarly, the R2 of the pruned genetic instruments finally used would be important to know.

If we understand the reviewer's concern correctly, they are concerned that the variance explained by the BMI and age of menarche by the lipid IVs is too low to accurately estimate the causal effects of these two exposures on breast cancer. We agree if only these IVs were used, it would be problematic. Instead, the multivariable MR method we used (which is presented in the two papers of Burgess et al 2015 in American Journal of Epi) does two steps. The first step estimates the effect of other exposures on the outcome using all significant SNPs for each exposure, and calculates the residuals. It then regresses the

residuals on the IVs of the exposure of interest (i.e. lipid trait) to estimate a corrected exposure/outcome effect. Since all significant BMI and age of menarche SNPs are used in step one, the instrument strength is significant.

Could genetic ancestry bias the results? The qqplots of the genetic instruments in the MVP study showed some inflation.

While the genomic control value is somewhat high after meta-analysis, we believe this elevated value results not from technical confounding or population stratification, but rather from an extensive, polygenic architecture of lipid traits, where most of that 'inflation' reflects a large number of weak genetic effects that remain to be discovered.

One way to evaluate this is by the use of LD score regression, to estimate the extent of inflation due to polygenicity vs. uncontrolled stratification from genetic ancestry mismatch (population structure) or other technical issues. The LD-score regression intercept captures the confounding effects of population stratification, and an intercept close to one indicates that the inflation in the summary statistics is due to pleiotropy, not confounding effects.

We perform this analysis and found that the LDSC intercepts and standard errors are as follows : Total Cholesterol: 1.1293 (0.1143), Triglycerides: 1.0317 (0.0656), LDL: 1.0933 (0.1001), HDL: 1.1715 (0.0758). All intercepts are within two standard errors of 1 except for HDL. However, the cross-trait LDSC intercept for HDL and Breast cancer-all is 0.0041 (0.0082), indicating that any possible confounding in the HDL summary statistics does not appear to be in the direction that would confound our analysis. Furthermore, if our MR results were due to the confounding effects of population stratification, one would expect that our Mendelian randomization results would differ between the GLGC and MVP cohorts. However, we found that estimates were reasonably consistent between the cohorts. In response to this comment, we have added the LD score intercepts to Supplementary Table 14 and have added population stratifications as one of the possible limitations of this approach in the "short-comings" sentence of the abstract suggested by the editor.

It is not explicit if the heterogeneity pruning was performed at the level of genotyping array, or at the country/population level, since each of the 3 BCAC subsets comprises multiple smaller studies performed in diverse populations.

We apologize for the confusion, and have modified our supplementary methods section to make our heterogeneity pruning more clear. Our heterogeneity pruning was to remove SNPs that have a much different ratio of effect between breast cancer and lipid than other SNPs. By doing this pruning, we are removing variants that have strong evidence for pleiotropic effects that may bias our causal effect estimates. We note that this is entirely different from removing SNPs showing differences in effects on breast cancer between different cohorts. We do not have access to the individual-level data, so are unable to perform any secondary analyses or filtering such as pruning for cohort heterogeneity. We have revised the supplementary methods sections to include the following:

Heterogeneity analyses for single trait MR

“Cochran’s Q test revealed significant heterogeneity in our genetic instruments’ ratios of effects on outcome and exposure in our single trait MR analyses (**Supplementary Table 5**). This heterogeneity suggests pleiotropic effects in the outlier SNPs. We constructed a second genetic instrument subjected to the following pruning procedure to ensure the genetic instruments for each lipid trait met the assumption of no significant heterogeneity of effects...”

Multivariable MR tests of instrument strength and validity

“As in single trait MR, heterogeneity of genetic instrument effects in multivariable MR can indicate pleiotropy, or invalid instruments.”

The discussion is very poor, with some emphasis on the potential adverse effects on increasing HDL by statins, but no discussion about the results of meta-analysis of cohort studies on BC risk that have measured plasma lipid levels.

In addition to adding a paragraph addressing the study of Beeghly-Fadiel et al. we have reworded a significant portion of our discussion to make it more precise and to-the-point. In addition, we have added the following sentence to the discussion section to address the meta-analyses of cohort studies:

"Previous meta-analyses of observational studies of breast cancer risk and lipids reported a negative association with HDL and no relationship with LDL [4,5], while individual studies have reported a positive relationship with HDL [6] or no relationship with HDL or LDL [42,43]. Our analyses help clarify these mixed results, and infer a direction of effect, which is not possible in observational studies due to potential reverse causation."

One of the interesting analysis performed is the gene-level approach. The finding of ABO and BRCA is intriguing. The epidemiological evidence of such association has been explored previously, with a possible higher risk of the A group, but the discussion is missing.

We have added additional details and discussion about our ABO finding, both to the results section and the discussion. Key to this discussion is the paper of Pickrell et al. 2017, which characterizes the eQTL and blood type associations at this locus. However, Pickrell et al did not find the breast cancer association at this locus. We have also added a citation for ABO blood type being associated with breast cancer.

Minor:
Abstract: end last sentence of Background

We have fixed this sentence.

Reviewer #4: GENERAL COMMENTS

Johnson et al. conduct a thorough and comprehensive MR analysis of lipid traits and breast cancer risk. The methods applied are appropriate and well described, and their conclusions reasonable given the results. My only major concern relates to the novelty of the study, given that MR analyses of lipid traits and breast cancer risk have previously been published, including a recent analysis by Beeghly-Fadiel et al. (IJE, 2019) (although it is important to note that the paper by Beeghly-Fadiel may have been published after submission of this paper by Johnson et al., I am not sure).

We appreciate the recognition of our methods and conclusions, and agree that our results overlap those of Beeghly-Faidel et al. As the reviewer points out, it is true that Beeghly-Fadiel et al was only recently published during the course in which our manuscript was under review at this journal (PLoS Medicine), evidenced by the pre-print of our manuscript we posted in late October prior to submitting here (https://www.biorxiv.org/content/10.1101/794594v3). As a result, we were not aware of the work during this initial review, and thus did not comment on it.

That now said, there are differences between the pieces of work, with complementary and detailed types of analyses presented in both. However, we believe that our work stands separately and the collection of both pieces of work provides a strong case for HDL as a potential risk factor.

We believe we address this point thoroughly in response to both this reviewer and Reviewer #5, below.

MAJOR COMMENTS

----

1) Beeghly-Fadiel et al (https://www.ncbi.nlm.nih.gov/pubmed/31872213) have recently published an MR analysis of lipid traits and breast cancer risk, using similar data sets and arriving at similar conclusions. The paper by Beeghly-Fadiel et al should be referenced, and given the similarity of the papers, a comprehensive comparison of methods employed and conclusions drawn should be made.

Beeghly-Fadiel et al is, of course, a nice contribution to the field. We now cite it and in our discussion we point out key differences between our manuscript and theirs, including the following five major differences:

1. We take advantage of genetic data from the MVP for lipid association discovery. This allows us to replicate prior MR analyses using an independent, higher-powered instrument.

2. Through our meta-analysis of both GLGC & MVP, we gain significantly higher powered lipid instruments than any previously available.

3. We perform conditional analyses at key HDL and LDL lipid loci. The genetic variants in this analysis were derived from a meta-analysis of MVP+GLGC summary statistics, which can not be immediately

extracted or inferred from the supplementary material presented in Beeghly-Fadiel et al. But more to the point, they do not present this style of analysis as we do here.

4. The multivariable analyses presented in the two manuscripts differ. Beeghly-Fadiel only considered each confounding factor (e.g. ER status or BMI) in their single lipid individual-level MR. Thus, their study contains no MR experiment that models multiple lipid traits and confounding factors jointly. In our manuscript, we include all these factors in our MVMR analysis. By doing so, we estimate the effect of each lipid on BC independent of all other factors. This proved useful, as in our multivariable experiments, which include adjustments for BMI, HDL, TG and Age-of-Menarche, we observed a positive association between elevated LDL and Breast Cancer risk (see Point 5).

5. In addition to a significant positive relationship between LDL and breast cancer risk in our MVMR analysis, we report a positive genetic correlation between all-cause breast cancer and LDL cholesterol levels. These findings distinguish our study from Beeghly-Fadiel, who emphasize that they found no relationship between LDL and breast cancer risk. Additionally, previous studies of the genetic correlation between lipids and breast cancer risk have not reported this relationship.

In reference to points (4) and (5) Beeghly-Fadiel found no association with LDL:

Page 6, col 1, line 5: “We found no association for LDL-C…”
Page 6, col 1, par. 2: “Regardless of adjustment, or whether initial or exclusive variants were included, the LDL-C wGS was not associated with breast cancer risk in our analyses.”
Parag. 1, discussion: “Genetically predicted LDL-C was not associated with breast cancer risk in any analyses.”

For these reasons our work not only provides a thorough confirmation of prior findings, but also contains multiple novel and complementary analyses.

MINOR COMMENTS
----

1) Line 10, "Randomization" is missing from the end of the sentence.

Corrected.

2) Line 109, authors should state whether they are using an additive or multiplicative random effects model.

We have added the relevant sentence in the Methods:
“Unless otherwise noted, MR results reported in this manuscript used inverse-variance weighting assuming a multiplicative random effects model.”

3) The authors should state how they harmonized the lipid and breast cancer GWAS SNPs (I assume it was using TwoSampleMR). They should clarify that they checked that all SNP-exposure association estimates and the SNP-outcome association estimates used the same effect alleles, and that there existed no palindromic or contradictory alleles.

We agree, and have now added to the methods section (line 154) that we used the harmonization function of TwoSampleMR.

4) Lines 173-174, the authors state that they also tested the relationship between lipids and breast cancer also using GLGC along. I assume this was as a sanity check of heterogeneity between the data sets, but this should be made clearer.

Indeed, this is exactly why we repeated our analysis using GLGC. We have added the below sentence:

"To confirm that our results using lipid genetic associations from MVP were not due to heterogeneity between datasets, we also tested the relationship between lipid traits and breast cancer using a meta-analysis of the two major lipid GWAS from MVP and GLGC, and GLGC alone."

5) The authors state that the meta-analysis of the Klarin and Miller GWAS "appears well-calibrated". However, the lambda values in Supplementary Figure 1 range from 1.29 to 1.51, indicating some inflation of the test statistics. The authors should state that such inflation exists, and discuss whether the inflation could affect the results of their MR analyses.

Indeed, these lambda-GC values are higher than in the GLGC or MVP-only GWASes. Population stratification (and other sources of confounding) and/or pleiotropy could be causing the inflation. But, in addition to that, an extensive, polygenic architecture of lipid traits, could also result in 'inflation' which reflects a large number of weak genetic effects that remain to be discovered.

One way to evaluate this is by the use of LD score regression, to estimate the extent of inflation due to polygenicity vs. uncontrolled stratification from genetic ancestry mismatch (population structure) or other technical issues. The LD-score regression intercept captures the confounding effects of population stratification, and an intercept close to one indicates that the inflation in the summary statistics is due to pleiotropy, not confounding effects.

We estimated the LDSC intercepts and standard errors as follows : Total Cholesterol: 1.1293 (0.1143), Triglycerides: 1.0317 (0.0656), LDL: 1.0933 (0.1001), HDL: 1.1715 (0.0758). All intercepts are within two standard errors of 1 except for HDL. However, the cross-trait LDSC intercept for HDL and Breast cancer-all is 0.0041 (0.0082), indicating that any possible confounding in the HDL summary statistics does not appear to be in the same direction. Furthermore, if our MR results were due to the confounding effects of population stratification, one would expect that our Mendelian randomization results would differ between the GLGC and MVP cohorts. However, we found that estimates were reasonably consistent between the cohorts. In response to this comment, we have added the LD score intercepts to

Supplementary Table 13 and have added population stratifications as one of the short-comings of this approach in the "short-comings" sentence of the abstract suggested by the editor.

6) How was the set of genes with protein products with key roles in HDL or LDL biology chosen? Is it from prior knowledge of the authors?

They were chosen using prior knowledge from the senior authors.

7) How evidence of directional pleiotropy was assessed should be described in the methods section.

Sentence added to methods:

We tested for directional pleiotropy using the MR-Egger regression test [25].

8) That causal effects were also estimated using MR-Egger, median-based, and mode-based approaches should be mentioned in the methods.

We have added this sentence to to the Methods:

For single trait MR analyses, we additionally employed Egger regression [24], weighted median [25] and mode-based [26] estimates.

9) Estimates of study power should be provided for each of the lipids. I assume that the powers for the null results are good, but it would be useful to have estimates presented anyway.

We are not aware of a method to estimate study power for multivariable, binary outcome, two-sample MR using summary statistics. For univarible MR for each of our lipid traits, we have estimated power based on PMID:24608958,25165093 for discovery for 80% power with an error rate of 1% to be (across all samples): HDL: 1.057, LDL: 1.058, TG: 1.055, TC: 1.060. We have included these numbers in the Methods section.

10) Line 163, triglycerides should be abbreviated for consistency.

We agree, and have changed this to "TG".

11) There have been a number of RCT of statins. Have any of these RCTs observed the predicted effects on breast cancer incidence?

There have been RCTs of statins, however these are primarily in men, and the study duration is not long enough to be able to measure an increase in breast cancer risk. As a response, we have added a citation for the sentence "These potential disease-increasing consequences may not have been possible to identify in safety trials, given the limited window of study to monitor progression or incidence of disease, the

putative causal effect estimates, and the demographics of the study population (*i.e.*, higher proportion of male participants) [52]. "

Alex Cornish (ICR, London). I am happy to have my name made available to the authors.

Reviewer #5: My main concern with the manuscript is that the headline findings have all already been published - based on the same or similar datasets and using Mendelian randomisation methods - and therefore this manuscript does not add anything new to the literature at all.

We appreciate the concern from this reviewer. Below, we articulate differences in the methodology, data and findings between our work and that of *Beeghly-Fadiel et al*.

In addition, we draw the reviewer's attention to the publication date of the *Beeghly-Fadiel et al* work, which is fairly recent (December, 2019). We had submitted our manuscript prior to this (we direct to a pre-print upload date of initial version of the manuscript, which was in mid October, https://www.biorxiv.org/content/10.1101/794594v3), and note that our work has been under review at Plos Med since that time.

We think that the diversity of analyses and data we present provides a novel and informative contribution to the field and this clinical question.

In this manuscript, Johnson et al reports an association between HDL cholesterol and risk for all breast cancers. However, the same association (overlapping confidence intervals and similar point estimates) has already been reported by Beeghly-Fadiel et al International Journal of Epidemiology 23 December 2019 (doi.org/10.1093/ije/dyz242) using the (nearly) identical Breast Cancer Association Consortium (BCAC) data set. Both Johnson et al and Beeghly-Fadiel et al report results taking into account the effect of body mass index, menopausal status, and estrogen receptor status. Both papers perform multivariable Mendelian randomisation analyses for all three lipid traits evaluated - LDL, HDL, TG.

Beeghly-Fadiel et al is, of course, a nice contribution to the field. We now cite it and in our discussion we point out key differences between our manuscript and theirs, including the following five major differences:

1. We take advantage of genetic data from the MVP for lipid association discovery. This allows us to replicate prior MR analyses using an independent, higher-powered instrument.

2. Through our meta-analysis of both GLGC & MVP, we gain significantly higher powered lipid instruments than any previously available.

3. We perform conditional analyses at key HDL and LDL lipid loci. The genetic variants in this analysis were derived from a meta-analysis of MVP+GLGC summary statistics, which can not be immediately extracted or inferred from the supplementary material presented in Beeghly-Fadiel et al. But more to the point, they do not present this style of analysis as we do here.

4. The multivariable analyses presented in the two manuscripts differ. Beeghly-Fadiel only considered each confounding factor (e.g. ER status or BMI) in their single lipid individual-level MR. Thus, their study contains no MR experiment that models multiple lipid traits and confounding factors jointly. In our manuscript, we include all these factors in our MVMR analysis. By doing so, we estimate the effect of each lipid on BC independent of all other factors. This proved useful, as in our multivariable experiments, which include adjustments for BMI, HDL, TG and Age-of-Menarche, we observed a positive association between elevated LDL and Breast Cancer risk (see Point 5).

5. In addition to a significant positive relationship between LDL and breast cancer risk in our MVMR analysis, we report a positive genetic correlation between all-cause breast cancer and LDL cholesterol levels. These findings distinguish our study from Beeghly-Fadiel, who emphasize that they found no relationship between LDL and breast cancer risk. Additionally, previous studies of the genetic correlation between lipids and breast cancer risk have not reported this relationship.

In reference to points (4) and (5) Beeghly-Fadiel found no association with LDL:

Page 6, col 1, line 5: “We found no association for LDL-C…”
Page 6, col 1, par. 2: “Regardless of adjustment, or whether initial or exclusive variants were included, the LDL-C wGS was not associated with breast cancer risk in our analyses.”
Parag. 1, discussion: “Genetically predicted LDL-C was not associated with breast cancer risk in any analyses.”

In this manuscript, Johnson et al also report genome-wide genetic correlation between cholesterol levels and breast cancer. However, these have already been reported by Jiang et al. Nature Communications 25 January 2019 using the same BCAC data set used in this manuscript (https://doi.org/10.1038/s41467-018-08054-4).

We thank the reviewer for pointing us to this previous literature, and we regret that we did not include the Jiang et al. 2019 citation in our initial submission. It is true that Jiang et al addresses the genetic relationship between circulating lipids and breast cancer risk (via genetic correlation). They utilized data from GLGC (Willer et al.), but report no significant genetic correlation with any lipid trait using LDSC (at $P < 0.01$) and all-cause breast cancer. Our approach and results differs here in the following ways:

1. We utilize the largest available lipids GWAS data on this question, i.e., the meta-analysis of Willer et al and MVP data to estimate genetic correlation. This corresponds to the highest powered lipid GWAS in the public domain. As a result we may be able to detect correlations that were too small to have been previously detected.

2. We utilize not only LDSC to perform genetic correlation analysis, but also an alternative method, ρ-Hess. One main difference between ρ-Hess and LDSC is that the ρ-Hess method uses a truncated singular-value decomposition regularization step to remove noise in external reference LD. The authors of that work demonstrated in simulation that their approach performs better with superior accuracy, and they conjecture that the regularization procedure is the main reason for this (reading carefully, this has to do with reducing the degrees of freedom required when estimating the covariances given the LD matrix). They note that their estimator provides lower standard errors as compared to cross-trait LDSC, likely due to the truncated-SVD regularization procedure. In addition, LDSC assumes a random effect for the SNP effect sizes, whereas ρ-Hess assumes a fixed effect model. In specific scenarios, the random effect sizes could also increase the estimated standard error relative to a fixed-effect model. The reduction in the standard error of covariance estimates directly relates to increased power for discovery of true genetic correlations, which may be why we make a positive discovery here for LDL and all-cause breast cancer (P = 0.0004), but was not observed in Jiang et al.

Thus, our positive genetic correlation results are novel.

Both Johnson et al and Beeghly-Fadiel et al report an inconsistent relationship between triglycerides and breast cancer risk.

We agree with the reviewer that the interpretations between the studies are correct on this point.

That said, we note that differences in the analytical approaches utilized between the two studies in terms of controlling for confounders and lipid traits jointly. By using MR which jointly considers Age of Menarche and BMI **and** other lipid traits, we argue that our failure to reject the null is of more clinical interest than a null finding which could just be due to these confounding factors. This multivariable approach was not used in Beeghly-Fadiel.

The only novel results presented by Johnson et al are gene-level analyses for HDL-associated genes (Figure 2) and local genetic correlation analyses that identify pleiotropic breast cancer risk- and LDL-associated SNPs near the ABO gene. However, this region is already known (published) to harbour genome-wide significant risk SNPs for LDL cholesterol and breast cancer risk in the publicly available data sets used by Johnson et al. The ABO locus itself is established as a highly pleiotropic region and this result is therefore not surprising (see for example: 10.1038/ng.3570).

We agree that it is perhaps unsurprising that ABO is associated with LDL and breast cancer, given that the ABO locus is highly pleiotropic. First off, we have now added a citation to 10.1038/ng.3570 to our discussion section as we agree with the reviewer that it helps put our finding in context. However, the application of local genetic correlation to these traits is still a novel analysis, even if the only significant locus is highly pleiotropic. We further note that other reviewers found this of interest, despite it being "known" from this reviewer's point of view; thus, highlighting this locus might be itself valuable given that the manuscript is about the genetic relationship between lipids and breast cancer.

Further, the results in Figure 2 of Johnson et al can be worked out using supplementary data from Beeghly-Fadiel et al.

The relevant supplementary data that appears to be referred (we think that is Supplementary Table 1) is simply the list of lead (sentinel) variants for each lipid trait association for each independent locus. From this data alone, we do not believe it is possible to identify conditionally independent variants located near these lead variants, as we did in the analysis shown in figure 2.

To perform this analysis, we use the conditional GWAS statistics from Klarin et al at these loci to identify SNPs independent of each lead association. These conditionally independent associations are then used in a locus-centric analysis, to calculate a locus-by-locus and combined estimate of the change-in-lipid level to change-in-breast cancer risk. This is a unique contribution and cannot be obtained and recapitulated from the supplementary data in *Beeghly-Fadiel* et al. We note that this is a particularly strong analysis, as these core lipid genes have a well-understood role in lipid biology, and thus have direct effects on lipid levels. Therefore these instruments are less susceptible to confounding due to pleiotropic effects than instruments with more poorly understood biology. Finally, these results provide direct causes for concern around potential therapeutic interventions. For example, the result at the *CETP* locus - a long sought-after target of therapeutics that elevate HDL-C for cardioprotection - suggests a negative outcome for CETP inhibition, one which may not have been detectable (or considered) in previous clinical trial work.

Finally, Johnson et al use cholesterol SNPs from the Million Veteran Program but the use of these MVP SNPs does not make any qualitative difference to the results compared to the use of the SNPs from the Global Lipid Genetics Consortium (used by Beeghly-Fadiel et al).

For HDL, in general, we do not disagree with the reviewer. As before, our paper presents a battery of complementary and unique multivariable experiments and a locus-centric analysis which certainly boost the credibility to our HDL findings.

For LDL, as discussed above, in our multivariable experiments of LDL **and** HDL and other confounders (BMI and age of menarche), we observed a modest, positive association between elevated LDL and Breast Cancer risk. Secondly, we report a positive genetic correlation between all-cause breast cancer and LDL cholesterol levels. These findings distinguish our study from Beeghly-Fadiel, who emphasize that they found no relationship between LDL and breast cancer risk.

For these reasons our work not only provides a thorough confirmation of prior findings, but also contains multiple novel and complementary analyses.
