# Supplementary material for: The relationship between circulating lipids and breast cancer risk: A Mendelian randomization study
Source: PLoS Med. 2020 Sep 11;17(9):e1003302. doi: 10.1371/journal.pmed.1003302 (PMC7485834; doi:10.1371/journal.pmed.1003302)
Supplement: Attachment — Submitted filename: Response to editor-round 2.docx [file pmed.1003302.s033.docx]

**Response to editors**

**Johnson & Siewert et al.**

**1. Abstract: Line 18: Please use either Mendelian randomization or the abbreviation MR consistently.**

We have changed to using the abbreviation "MR".

**2. Abstract: Line 29: Please revise this sentence to avoid implications of causality: “In addition, we found evidence that genetic variation at the ABO locus is associated with both lipid levels and breast cancer” or similar.**

We have changed this to "variation at the ABO locus is associated with both lipid levels and breast cancer".

**3. Abstract: Conclusions: Line 35: Would it make sense here to also mention the association between LDL and breast cancer risk that you identified with multivariable MR? (mentioned above at Line 22-23)?**

Yes, we originally only mentioned HDL because the confidence interval for LDL in the single-trait MR included 1. However, given that the direction of effect is the same between the single and multivariable MR, and that the single trait MR p-values are very close to 0.05, we have now added mention of the LDL finding:

"We observed that genetically elevated plasma HDL and LDL levels appear to be associated with increased breast cancer risk. Future studies are required to understand the mechanism underlying this putative causal relationship, with the goal to develop potential therapeutic strategies aimed at altering the cholesterol-mediated effect on breast cancer risk."

**4. Author Summary: Thank you for providing an author summary. Under “Why was this study done?” we suggest that you combine the first two bullet points. At line 35, under the third bullet point, we suggest changing “genetics” to “Mendelian randomization methods” or similar to enhance specificity.**

We have made both these changes as suggested.

**5. Author Summary: Please use the first person perspective. For example, please use “We tested…” at line 49.**

We have switched to using first person throughout the author summary (lines 49 and 51).

**6. Under “What do these findings mean?” Please delete the first and third bullet point, and replace it with a sentence that can address the study implications without overreaching what can be concluded from the data; we suggest: "Further research will be needed to investigate the possibility that manipulation of LDL or HDL levels can influence risk of breast cancer"**

We agree, and have changed these sentences to those suggested by the editor

**7. Introduction: Line 93: Rather than "nominal", please change to "association of uncertain significance" or similar, to clarify.**

We agree this was originally ambiguous. We have now changed the wording to use the term "borderline statistically significant'', to highlight that the finding could be regarded as significant, which is what Qi & Chatterjee use when referring to this finding. We say:

"Another recent study by Qi & Chatterjee applied a newly developed MR method and reported an association between HDL-cholesterol and breast cancer that they defined as borderline statistically significant"[13].

**8. Introduction: Line 101-105: This would be more appropriate in the Discussion section: “A recent study by Beeghly-Fadiel et al., published following the submission of this manuscript, performed an MR analysis of breast cancer risk that considered potentially confounding risk factors and draws some of the same major conclusions as we do [21]. We highlight the distinctions between that study and the present manuscript in the Discussion.”**

We agree. We have deleted this passage from our introduction and merged it into the relevant section of our discussion (lines 386-387), saying:

"Our results largely agree with those reported in the recent MR study of breast cancer and lipids by Beegly-Fadiel et al, published following the submission of this manuscript."

**9. Methods: Lines 133-135: Please provide details on the ethical approval/name the specific University IRBs that provided approval. Please specify whether informed consent was written.**

We have added the following to this section: "The MVP received ethical and study protocol approval from the Veteran Affair Central Institutional Review Board in accordance with the principles outlined in the Declaration of Helsinki, and written consent was obtained from all participants. For the Willer et al and BCAC datasets, we refer the reader the primary GWAS manuscripts and their supplementary material for details on consent protocols for each of their respective cohorts." (Lines 142-145)

**10. Discussion: Lines 345-346: We suggest revising this sentence to read: “Using Mendelian randomization, we provide evidence that genetically elevated HDL and LDL levels are associated with increased risk for breast cancer…”**

We have changed this to the suggested wording. (Lines 360-362)

**11. Discussion: Lines 371-372: We suggest not abbreviating Beeghly-Fadiel et al. “(hereafter B-F)” as this is only used once in the paragraph, and could cause confusion.**

Agreed, we have now changed B-F to Beeghly-Fadiel et al. (Paragraph starting at line 386)

**12. Checklist: Please ensure that the study is reported according to the STROBE guideline, and include the completed STROBE checklist as Supporting Information (or, please report your study according to the relevant guideline, which can be found here:** [**http://www.equator-network.org/**](http://www.equator-network.org/)**)**

**When completing the checklist, please use section and paragraph numbers, rather than page numbers.**

**Please add the following statement, or similar, to the Methods: "This study is reported as per the Strengthening the Reporting of Observational Studies in Epidemiology (STROBE) guideline (S1 Checklist)."**

We have added this sentence to line 221 and provide a completed STROBE-MR checklist in our supplementary material.

**13. Supplementary Tables: Please provide titles and legends for each individual table and figure in the Supporting Information, rather than including these as a list in another supporting information file.**

We have now included the caption for each supplementary table on the first line of the table. When we attempted to upload each supplementary table individually to the submission system and pasting the caption into the "Description" field, it cut off our file descriptions. It is unclear to us how to best include supplementary table captions/legends, and would be happy to change how we are including them if given further instructions.

**Comments from the Academic Editor:**

**This paper uses Mendelian randomization to assess the causal effects of lipids on breast cancer, but a much stronger case (in Introduction and Discussion) needs to be made to clarify what it adds to the existing knowledge from similar MR studies. The analyses reported are very comprehensive, and yet the rationale and practical implications of the different approaches used is not always clear to the reader – which makes it difficult to judge its added value compared with previous MR work.**

**For example, the authors don’t discuss the implications of using a gene-specific vs. a conventional MR analysis, or a local vs. genome-wide genetic correlation analysis, and why using a gene-specific MR or a local genetic correlation analysis is important. Moreover, the MR and genetic correlation approaches answer different questions, and yet this is not clearly discussed.**

We thank the editor for calling this to our attention and we agree that adding significantly more explanation of our approaches and their strengths is important. We have now added the following explanation of the advantages of our gene-specific approach (line 404) to the Discussion:

"A challenge of MR analyses that use hundreds of genetic instruments is that we do not know the mechanism of action of these instruments on the exposure trait. By focusing on loci with mechanistic evidence of a direct effect on lipid levels, we can remove uncertainty about potential pleiotropic effects on breast cancer risk. Thus, the significant relationships observed in our locus-specific MR analyses across HDL or LDL pathway genes provides additional evidence for a direct effect of increased HDL or LDL levels on breast cancer risk. Furthermore, these genes represent potential or established drug targets, and each locus-specific MR experiment provides preliminary evidence for the therapeutic potential of cholesterol modification on breast cancer prevention.."

We have also added an explanation of the importance of these methods to the Introduction (Line 120):

"Of chief concern in modern MR studies, including prior studies of breast cancer and lipids, is confounding due to pleiotropy. For instance, a genetic variant may affect lipid levels indirectly through some other biomarker. If this biomarker directly affects breast cancer risk, this could confound the MR analysis and cause an incorrect inference of a causal effect of lipid levels on breast cancer. Our gene-specific approach utilizes only genetic variants near core HDL pathway genes to minimize this concern. Additionally, we use a multivariable approach that assesses the effects of lipid traits independent of one another and of BMI and age at menarche."

In addition, we now explain the differences and advantages between MR, genome-wide, and local genetic correlation in the introduction (lines 103-107):

"While MR assesses evidence for a causal relationship, genome-wide genetic correlation analysis tests whether two traits simply have a shared genetic basis. Local genetic correlation analyses test whether two traits have shared heritability that is localized to specific genomic regions. These loci may then harbor causal variants and genes that contribute to heritability of both traits."

**Regarding the MR methods used, I think some justification (or reference to a method paper) is required for the method used to perform the “Heterogeneity analyses for single trait MR” described in the Supplement. Similarly, more justification is needed for the pruning used in the multivariable analysis (“stepwise post-hoc procedure to remove genetic instruments that contributed the most to QA”).**

The approach of using Cochran’s Q to test for heterogeneity in single-trait MR is described in reference 4 of the Supplementary Methods (Bowden et al. 2018)-- we had included this citation later in this section but have now added it to the following sentence, along with a citation for another paper that discusses the pros and cons of this approach (Bowden et al. 2019). We also have added that we performed this test with estimates from inverse-variance weighted MR:

“Cochran’s Q revealed significant heterogeneity in the genetic instruments’ ratios of effects on outcome and exposure in our initial single trait inverse-variance-weighted MR analyses (**Supplementary Table 5**) [4,5].”

We have also added the following discussing the assumptions of using Cochran’s Q to assess heterogeneity to the Supplementary material, and justifying our removal of heterogeneous instruments:

“Using Cochran’s Q with inverse-variance weights makes the NOME (no measurement error assumption), which can become problematic in the presence of weak genetic instruments [5]. Due to the large GWAS sample size and significant heritability of the lipid traits used here, strong measurement error or weak instruments are not significant concerns and as a result Cochran’s Q is an appropriate test of heterogeneity. By removing potentially pleiotropic variants from our single-trait and multivariable MR analyses (see below), we better satisfy the assumptions of causal inference via MR [6]. Importantly, we note that the estimates from our MR analyses before and after pruning for instrument heterogeneity are in strong agreement.”

We added the following to the methods as well (line 177):

“Because MR assumes homogeneity between each instrument’s exposure to outcome effect, we then tested for instrument strength and validity [35], ...”

**The tests of instrument strength and validity reported in the Supplement also require some mention to correlation assumptions when applied to two-sample MR.**

We believe the editor is referring to the following (from Sanderson et al. 2019): “If the covariance between the effect of the genetic variants on each exposure can be either: (i) estimated from individual data, (ii) assumed to be zero or (ii) fixed at zero by using non-overlapping samples for each exposure genome-wide association study (GWAS), then our proposed summary data Q statistics will give a good approximation of the true (individual-level data) result.”

To address this point, we have added the following to the **Supplementary Methods**:

To calculate Q_X1_ and Q_A_, the covariance between the genetic instruments’ effects on each exposure must be estimated from individual-level data or assumed to be zero (Sanderson et al. 2019). As we did not have access to individual-level data, we assumed this covariance term was zero. To test the effect of this assumption, we also performed multivariable MR using non-overlapping samples (using MVP paired with GLGC) for each of the lipid exposure traits (Supplementary Figure 7, Supplementary Table 11), thus fixing covariance at zero.

**Comments from the reviewers:**

**Reviewer #4: The authors have addressed my comments.**

**Alex Cornish (ICR, London).**
