# Supplementary material for: The relationship between circulating lipids and breast cancer risk: A Mendelian randomization study
Source: PLoS Med. 2020 Sep 11;17(9):e1003302. doi: 10.1371/journal.pmed.1003302 (PMC7485834; doi:10.1371/journal.pmed.1003302)
Supplement: Attachment — Submitted filename: Response_to_editors_08052020.docx [file pmed.1003302.s034.docx]

"The relationship between circulating lipids and breast cancer risk: a Mendelian randomization study" (PMEDICINE-D-19-03886R3)

Johnson, Siewert, et al.

Response to editors’ comments

Due date: ASAP

**## Overview**

**In providing this most recent round of uploading (and renaming files), the Editorial Manager Website was bugged – for the supporting information, it changed the file name(s) to “lower case” for the “Fig” or “Table”; from “S1_Fig.pdf” to “S1_fig.pdf”. I waited a day to see if the website would ‘fix’ itself but it would not, so this wasn’t something I could fix. I suspect that this is due to the fact that on previous versions, there’s a similar file name in the system previously uploaded, but that’s complete speculation.**

Requests from Editors and the Academic Editor:

1. Please indicate the corresponding author on the title page with an asterisk (*) and include the corresponding author's email address. Only an asterisk and the email address itself are required.

**Done.**

2. Please ensure that all Supporting Information files are clearly labeled in the file inventory as follows, using underscores: S1_Fig.tif, S1_Table.xlsx, S1_Text.doc, S1_Data.xlsx, S1_PRISMA_Checklist.docx, etc.

**Done.**

Each file type should be numbered starting at 1. Please also make sure that the file description matches the file name (e.g. S1_Fig.tif is listed as S1 Fig).

**Confirmed.**

3. Please label your main figure files as follows: Fig1.tif, Fig2.tif etc. Do not use underscores or spaces.

**Done.**

4. Before resubmitting your paper, please ensure that all files referenced in the manuscript are present in the File Inventory, as missing files will result in further delays. Please also ensure that all file names adhere to our naming conventions and that all in-text citations and supporting information file legends/captions do as well.

**We have confirmed this (again).**
